# FGFR1 Suppresses STING-Mediated Interferon Response in Endocrine Therapy–Resistant Breast Cancer

**DOI:** 10.1158/2767-9764.CRC-25-0821

**Published:** 2026-09-21

**Authors:** Torbjørn Amundsen Lien, Sushil Dhakal, Anne Marthe Fosdahl Wium, Helga Bergholtz, Serhat Gunesten, Helene Midtun Flatekvål, Daniel Nebdal, Phuong Vu, Antoni Hurtado, Jens Henrik Norum, Therese Sørlie

**Affiliations:** 1 Department of Cancer Genetics, Institute for Cancer Research, Oslo University Hospital, Oslo, Norway.; 2 Functional Cancer Genomics Group, Centro de Investigación del Cáncer, Instituto de Biología Molecular y Celular del Cáncer, Consejo Superior de Investigaciones Científicas, Universidad de Salamanca, Salamanca, Spain.; 3 Institute of Clinical Medicine, Faculty of Medicine, University of Oslo, Oslo, Norway.

## Abstract

Fibroblast growth factor receptor 1 (*FGFR1*) amplification is frequently observed in estrogen receptor–positive (ER^+^)/human epidermal growth factor receptor 2–negative (HER2^−^) breast cancer and has been linked to poor response to endocrine therapy. Although FGFR1 has been implicated in therapeutic resistance, its role in regulating interferon (IFN) response in the context of endocrine resistance remains unclear. We investigated whether FGFR1 modulates the IFN response through the cyclic GMP–AMP synthase (cGAS)–stimulator of IFN genes (STING) pathway in tamoxifen-resistant breast cancer. We employed tamoxifen-resistant ER^+^ breast cancer cell lines and a tamoxifen-sensitive *FGFR1*-amplified patient-derived xenograft (PDX) model to examine the impact of FGFR1 inhibition on the IFN response through RNA sequencing, FGFR1 knockdown or pharmacologic inhibition, and functional assays. Clinical relevance was evaluated in breast cancer cohorts. Tamoxifen-resistant cells exhibited increased cytosolic, cGAS-positive double-stranded DNA (dsDNA) foci alongside elevated phosphorylated IRF3 (p-IRF3), indicating constitutive engagement of the cGAS–STING pathway. FGFR1 inhibition resensitized resistant cells to tamoxifen and induced IFN response gene expression, which was further amplified by tamoxifen cotreatment. FGFR1 inhibition similarly potentiated tamoxifen-induced IFN response gene expression *in vivo* in an *FGFR1*-amplified PDX model. Mechanistically, FGFR1 knockdown enhanced STING-mediated *IFNB1* expression in response to cytosolic DNA. In the Molecular Taxonomy of Breast Cancer International Consortium (METABRIC) breast cancer cohort, *FGFR1* amplification was associated with poor prognosis specifically in tumors with high *STING1* expression. These findings identify FGFR1 as a suppressor of cGAS–STING–mediated IFN response, driven by tamoxifen in ER^+^ breast cancer. Attenuation of this response may represent a previously unrecognized contribution of FGFR1 to tamoxifen resistance.

**Significance:**

We identify FGFR1 as a suppressor of tamoxifen-driven IFN response in ER^+^ breast cancer and propose that attenuation of this response contributes to endocrine resistance. Our findings suggest that *FGFR1* amplification combined with high *STING1* expression could serve as a prognostic biomarker in trials of FGFR1-targeted therapies.

## Introduction

Estrogen receptor–positive/human epidermal growth factor receptor 2–negative (ER^+^/HER2^−^) breast cancer accounts for approximately 70% of all breast cancers (1). Although endocrine therapies such as tamoxifen significantly improve patient outcomes, acquired resistance remains a major clinical challenge, often leading to disease recurrence. Tamoxifen treatment has been associated with an interferon (IFN) response, described both as a direct result of ER inhibition and as an off-target effect of tamoxifen-induced DNA damage (2–5). IFNs are key regulators of immune activation and tumor-intrinsic stress responses, playing an important role in cellular defenses against DNA damage and other oncogenic stressors. Activation of IFNs leads to expression of a wide array of downstream effector proteins commonly referred to as IFN-stimulated genes (ISG; reviewed in ref. 6). Although ISGs are increasingly recognized as being associated with both tamoxifen response and resistance (7), the regulatory mechanisms underlying the IFN response in endocrine resistance remain poorly understood. Emerging evidence suggests that oncogenic signaling pathways, including those mediated by receptor tyrosine kinases, may intersect with IFN signaling to modulate ISG expression (8, 9). Among these, fibroblast growth factor receptor 1 (*FGFR1*) is frequently amplified in ER^+^/HER2^−^ tumors and has long been linked to endocrine resistance and poor prognosis, making it a potential therapeutic target (10–12). However, despite promising preclinical evidence, clinical trials of targeted FGFR therapies have demonstrated only modest efficacy in breast cancer (13–15), highlighting a need for biomarker refinement to better predict treatment response and improve patient stratification. Evidence suggests that FGFR1 may also influence innate immune signaling and the IFN response (8, 16). The mechanisms underlying this cross-talk are still poorly understood. In this study, we highlight an association between FGFR1 and the cyclic GMP–AMP synthase (cGAS)–stimulator of IFN genes (STING) axis, a DNA-sensing pathway that activates type I IFN signaling in response to cytosolic DNA (17–19). STING is a central regulator of the IFN response and has gained particular attention in the context of therapy resistance (20). We identify FGFR1 as a previously unrecognized modulator of STING-mediated IFN response in endocrine-resistant breast cancer. These findings suggest a broader immunoregulatory role for FGFR1 and highlight the need to refine predictive biomarker strategies by incorporating IFN-related pathways that may shape its function in endocrine resistance.

## Materials and Methods

### Cell culture and transfections

The tamoxifen-resistant T47D cell line (TR-T47D, RRID: CVCL_1D36) and its parental cell line (P-T47D, RRID: CVCL_1D45) and the tamoxifen-resistant MCF7 cell line (TR-MCF7, RRID: CVCL_M436) were a kind gift from Jan Stenvang (University of Copenhagen, Denmark). Wild-type (WT) MCF7 cells (RRID: CVCL_0031) were obtained from ATCC. These cell lines were authenticated by short tandem repeat profiling. TR-T47D and TR-MCF7 cells were generated by long-term treatment with 1 μmol/L tamoxifen as previously described (21). TR-T47D cells were cultured in phenol red–free RPMI 1640 medium (Gibco, cat. #11835030) supplemented with 2% charcoal-stripped fetal bovine serum (CSFBS, Gibco, cat. #12676029), GlutaMAX (2 mmol/L, Gibco, cat. #35050061), insulin (8 μg/mL, Sigma-Aldrich, cat. #I9278), and 4-hydroxytamoxifen (4-OHT; 1 μmol/L, Sigma-Aldrich, cat. #H6278). P-T47D cells were cultured in RPMI 1640 medium (Gibco, cat. #21875034) supplemented with 10% fetal bovine serum (FBS; Gibco, cat. #A5256801), GlutaMAX (2 mmol/L), and insulin (8 μg/mL). TR-MCF7 cells were cultured in phenol red–free DMEM/F-12 medium (Gibco, cat. #21041033) supplemented with 1% FBS, GlutaMAX (2 mmol/L), insulin (6 μg/mL), and 4-OHT. *FGFR1*-overexpressing MCF7 cells (R1-MCF7, RRID: CVCL_B5PF) were a gift from Jørgen Wesche (Institute for Cancer Research, Oslo, Norway; ref. 22). WT-MCF7 and R1-MCF7 cells were cultured in DMEM (Gibco, cat. #41966029) supplemented with 10% FBS and GlutaMAX (2 mmol/L). For pharmacologic inhibition of FGFR1, cells were treated with 5 μmol/L erdafitinib (JNJ-42756493, Selleck Chemicals, cat. #S8401). Stable *FGFR1* knockdown and control cells (TR-T47D^shFGFR1^, TR-T47D^shCTRL^, TR-MCF7^shFGFR1^, and TR-MCF7^shCTRL^) were generated by transduction with MISSION short hairpin RNA (shRNA) lentiviral particles targeting *FGFR1* (Sigma-Aldrich, cat. #TRCN0000121185) and MISSION pLKO.1-puro Non-Mammalian shRNA Control Transduction Particles (Sigma-Aldrich, cat. #SHC002V). Cells were transfected with lentiviral particles in polybrene infection/transfection reagent (Sigma-Aldrich, cat. #TR-1003) according to the manufacturer’s protocol. The cells were selected with 2 μg/mL of puromycin (Sigma-Aldrich, cat. #P4512) after 48 hours of treatment with lentiviral particles. *FGFR1* knockdown was confirmed using quantitative real-time PCR (qRT-PCR). TR-T47D^shCTRL/shFGFR1^ and TR-MCF7^shCTRL/shFGFR1^ cells were cultured in RPMI 1640 medium supplemented with 10% FBS, GlutaMAX (2 mmol/L), insulin (8 μg/mL), and maintained in 2 μg/mL puromycin to ensure stable shRNA expression. For poly(dA:dT) transfections, cells were incubated with 5 μg/mL precomplexed Poly(dA:dT)/LyoVec (InvivoGen, cat. #tlrl-patc) for 24 hours, with H_2_O used as a control. Cell lines were regularly tested for *Mycoplasma* infection using the MycoAlert Mycoplasma Detection kit (Lonza, cat. #LZ-LT07-218) and never kept in continuous culture for more than 13 passages after thawing.

### Protein isolation and Western blotting

Cells were lysed on ice in sodium dodecyl sulfate (SDS) lysis buffer (10 mmol/L Tris, pH 8, 1 mmol/L ethylenediaminetetraacetic acid, and 1% SDS) supplemented with 1:100 Halt Protease and Phosphatase Inhibitor Cocktail (Thermo Fisher Scientific, cat. #78440). The protein concentration was measured using a BCA Protein Assay Kit (Thermo Fisher Scientific, cat. #23227). Lysates were homogenized using QIAshredder columns (Qiagen, cat. #79656) and NuPAGE lithium dodecyl sulfate (LDS) Sample Buffer (4X; Thermo Fisher Scientific, cat. #NP0007), supplemented with dithiothreitol (DTT; 1:20). Lysates were run on 4% to 20% Mini-PROTEAN TGX Precast Protein Gels (Bio-Rad, cat. #4561094) in Tris/glycine/SDS buffer (Bio-Rad, cat. #1610732). Gels were blotted onto Trans-Blot Turbo polyvinylidene difluoride membranes (Bio-Rad, cat. #1704156/#1704157) using a Trans-Blot Turbo Semi-Dry Transfer System (Bio-Rad). Membranes were blocked with 5% w/v bovine serum albumin (BSA, Sigma-Aldrich, cat. #A9418) in Tris-buffered saline (Bio-Rad, cat. #1706435) and Tween 20 (Bio-Rad, cat. #1610781). Anti-FGFR1 (Cell Signaling Technology, cat. #9740, RRID: AB_11178519), anti-STING (Cell Signaling Technology, cat. #13647, RRID: AB_2732796), anti-IRF3 (Cell Signaling Technology, cat. #4302, RRID: AB_1904036), anti–phosphorylated IRF3 (p-IRF3; Cell Signaling Technology, cat. #4947, RRID: AB_823547), anti–β-actin (Santa Cruz Biotechnology, cat. #sc-47778, RRID: AB_626632), and anti–glyceraldehyde phosphate dehydrogenase (GAPDH; Abcam, cat. #ab181602, RRID: AB_2630358) were used as primary antibodies. Horseradish peroxidase–conjugated goat anti–rabbit IgG (Thermo Fisher Scientific, cat. #31460, RRID: AB_228341) and anti–mouse IgG (Dako, cat. #P0447, RRID: AB_2617137) were used as secondary antibodies.

### Immunocytochemistry and image quantification

Cells were seeded onto sterile glass coverslips and allowed to attach prior to treatment. Coverslips were rinsed 1× in PBS and fixed in 10% formalin (15 minutes, room temperature), washed 2× in PBS, and stored in PBS at 4°C until use. Coverslips were permeabilized in 0.2% Triton X-100/PBS (5 minutes) and washed 3× in PBS. Mouse anti–double-stranded DNA (dsDNA; 1:1,000; Abcam, cat. #ab27156, RRID: AB_470907) and rabbit anti-cGAS (1:200; Cell Signaling Technology, cat. #15102, RRID: AB_2732795) were applied in complete DMEM (1 hour, room temperature), followed by 3× washes in PBS. Secondary antibodies, goat anti–mouse IgG conjugated with Alexa Fluor 647 (1:1,000; Thermo Fisher Scientific, cat. #A21236, RRID: AB_2535805) and donkey anti–rabbit IgG conjugated with Alexa Fluor 488 (1:1,000; Thermo Fisher Scientific, cat. #A21206, RRID: AB_2535792), were applied in complete DMEM (1 hour, room temperature, dark), followed by 3× washes in PBS. Nuclei were counterstained with Hoechst 33258 (1:1,000, 5 minutes) and washed 3× in PBS. Coverslips were rinsed in distilled water, air-dried, and mounted on glass slides with Mowiol. Images were acquired on a Zeiss LSM 880 confocal microscope (Advanced Light Microscopy Core Facility, Montebello node, Institute for Cancer Research, Oslo University Hospital) with a 63×/1.4 numerical aperture oil-immersion objective (Plan-Apochromat) and processed using ZEN Black software (Zeiss, RRID: SCR_018163). Image analysis was performed using ImageJ (RRID: SCR_003070; ref. 23). Nuclei were identified by thresholding the 4′,6-diamidino-2-phenylindole (DAPI) channel to generate a nuclear mask. This mask was used to quantify the nuclear localization of cGAS by measuring the fluorescence intensity within the segmented nuclear regions. To quantify the nonnuclear distribution of dsDNA and cGAS, positively stained dsDNA regions were identified by intensity thresholding to generate a dsDNA mask. The nuclear mask was subtracted from the dsDNA mask, resulting in a nonnuclear dsDNA-positive region of interest. The number of nonnuclear dsDNA foci was normalized to the number of nuclei per image. A fixed intensity threshold, defined as the 95th percentile of mean cGAS intensity across all P-T47D foci, was applied uniformly across all images to classify foci as cGAS positive. Fluorescence intensities of both dsDNA and cGAS within these nonnuclear regions were then quantified. Thresholding parameters were kept consistent across samples. Statistical comparisons were performed using the Wilcoxon rank-sum test.

### Cell proliferation assays

Cell proliferation assays were performed using live cell imaging and Incucyte ZOOM or Incucyte S3 (Sartorius). Cells were seeded in 96-well microplates at 5% to 15% confluency. All cells were serum-starved for 48 hours in phenol red–free RPMI 1640 medium supplemented with 2% CSFBS, GlutaMAX (2 mmol/L), and insulin (8 μg/mL) prior to treatment with vehicle [0.05% DMSO (Sigma-Aldrich, cat. #D2650)], 4-OHT (1 μmol/L), erdafitinib (5 μmol/L), and erdafitinib (5 μmol/L) + 4-OHT (1 μmol/L) in fresh medium. All cells were analyzed by phase-contrast microscopy imaging at 3-hour intervals from the start of serum starvation up to 120 hours after treatment. Confluence values were normalized first to baseline (T = 0 hours) for each well and subsequently to the time-matched DMSO control average within each experiment. Statistical analysis was performed using a linear mixed-effect model fitted using the lme4 package (version 1.1-35.5, RRID: SCR_015654), with treatment condition as a fixed effect and biological replicate as a random intercept. *Post hoc* pairwise comparisons were conducted using the emmeans package (version 1.10.4, RRID: SCR_018734), with *P* values adjusted using the Tukey method.

### Cell viability assays

Cells of each cell line (P-T47D, TR-T47D^shCTRL^, and TR-T47D^shFGFR1^) were seeded at 5,000 cells per well in a 96-well plate in RPMI 1640 medium supplemented with 10% FBS, insulin (8 μg/mL), and penicillin–streptomycin (Sigma-Aldrich, cat. #P0781). The cells were treated the following day with DMSO (0.1%) or 4-OHT (0.001–10 μmol/L) for 120 hours. Cell viability was measured using CellTiter 96 AQueous One Solution (Promega) according to the manufacturer’s protocol.

### RNA isolation

Total RNA was isolated with the RNeasy isolation kit (Qiagen) according to the manufacturer’s protocol. A NanoDrop 2000 spectrophotometer (Thermo Fisher Scientific), a TapeStation system (Agilent), and a Qubit fluorometer (Thermo Fisher Scientific) were used to assess the quality and quantity of extracted RNA.

### qRT-PCR

RNA was extracted using the RNeasy Mini Kit (Qiagen, cat. #74104), and 1 μg of total RNA was reverse-transcribed using the High-Capacity cDNA Reverse Transcription Kit (Applied Biosystems, cat. #4368814). qRT-PCR was performed on a QuantStudio 7 Pro Real-Time PCR System using TaqMan Gene Expression Assays (Thermo Fisher Scientific) according to the manufacturer’s instructions. The following assays were used: *RSAD2* (Hs00369813_m1), *MX1* (Hs00895608_m1), *OAS1* (Hs00973635_m1), *IFNA2* (Hs00265051_s1), *IFNB1* (Hs01077958_s1), *TMEM173/STING1* (Hs00736955_g1), *FGFR1* (Hs00241111_m1), *ACTB* (Hs01060665_g1), *GAPDH* (Hs02786624_g1), *RPLP1* (Hs02926887_g1), and *HPRT1* (Hs02800695_m1). Gene expression was normalized using the ΔCq method relative to the indicated housekeeping genes. The same housekeeping gene pair was consistently used within each comparison for ΔΔCq and fold-change (FC) calculations. Pairwise comparisons of ΔCq values were performed using the Welch *t* test followed by Benjamini–Hochberg correction for multiple testing. FCs were calculated using the ΔΔCq method. A minimum of three biological replicates were included per condition.

### RNA sequencing and data processing

RNA sequencing (RNA-seq) was performed separately on three datasets: dataset 1, comprising P-T47D and TR-T47D cell lines; dataset 2, comprising MAS98.06 patient-derived xenograft (PDX) tumors; and dataset 3, comprising TR-T47D^shFGFR1^ and TR-T47D^shCTRL^ cell lines. Sequencing, library preparation, read processing, and count quantification for datasets 1 and 2 were performed by the Norwegian Sequencing Centre (University of Oslo, Norway); the same steps for dataset 3 were performed by Novogene. Full details of preprocessing and downstream analysis pipelines are available at https://github.com/taomli/Future_FGFR1_STING.

### RNA-seq data analyses

All downstream analyses of RNA-seq data, including normalization, differential gene expression analysis (DGEA), and gene set enrichment, were performed using the DESeq2 R package (v1.28.1, RRID: SCR_015687; ref. 24). Differential gene expression was assessed using the Wald test implemented in DESeq2. Genes with a Benjamini–Hochberg–adjusted *P* value (FDR) ≤ 0.05 were considered significantly differentially expressed. Gene set enrichment analysis (GSEA) was performed using the R package fgsea (v.1.14.0, RRID: SCR_020938; bioRxiv 2016.06.20.060012). Genes were ranked based on log_2_ FC values resulting from DGEAs. Gene sets were downloaded from the Molecular Signatures Database (MSigDB; v7.4, RRID: SCR_016863). A Benjamini–Hochberg–adjusted *P* value (FDR) of 0.05 or less was used as the threshold for calling significantly enriched gene signatures.

### PDX models and tumor growth measurements

For *in vivo* experiments, a previously established *FGFR1*-amplified, ER^+^/HER2^−^ PDX model (MAS98.06) was used (25). The previously established *FGFR1* nonamplified ER^+^/HER2^−^ PDX model HBCx34 was used to compare protein levels of FGFR1 in MAS98.06 (26). MAS98.06 PDX tumors were propagated by serial transplantation of tumor tissue (2–3 mm^3^) directly into mammary fat pad number 4 on both sides of 4- to 8-week-old female nonobese diabetic/severe combined immunodeficient (NOD/SCID) interleukin 2 receptor gamma chain null (Il2rg^−/−^; NSG) mice under general anesthesia. The MAS98.06 PDX tumor is dependent on estrogen supplements for growth, and 17-β-estradiol was administered in the drinking water. Tumor diameters (d_min_ and d_max_) were measured twice weekly using a caliper, and tumor volume was calculated using the formula ${d_{\min}}^{2}\cdot d_{\max}\cdot \frac{\pi }{6}$. The total tumor volume per animal was calculated by summing the tumor volume from both sides. Drugs were administered when the tumor volume reached approximately 200 mm^3^. Mice were randomized into four treatment groups: control (*n* = 13), tamoxifen (*n* = 7), erdafitinib (*n* = 5), and tamoxifen + erdafitinib (*n* = 5). Tamoxifen was administered by subcutaneous implantation of 60-day release pellets (Innovative Research of America), erdafitinib (12.5 mg/kg) was administered daily by oral gavage, and treatment was carried out for 27 days. The relative total tumor volume was calculated by dividing the measured individual tumor volume by the tumor volume at the treatment start. Treatment groups were compared at day 27 using a rank-based analysis of covariance (ANCOVA), adjusting for tumor volume at treatment start as a covariate. Pairwise comparisons were conducted using the emmeans package with Benjamini–Hochberg correction. All animal experiments were performed under institutional and governmental ethical oversight and were approved by The Norwegian Food Safety Authority (approval number: 13711).

### Protein isolation from PDX tumors

Protein was extracted from PDX tumor tissue using the Qproteome Mammalian Protein Prep Kit (Qiagen) according to the manufacturer’s recommendations. Fresh frozen tumor tissue (40 mg) was homogenized on ice using steel beads and a TissueRuptor homogenizer (Qiagen), and cells were lysed in 1 mL mammalian cell lysis buffer supplemented with Halt protease and phosphatase inhibitor cocktail. The protein concentration was measured using the BCA Protein Assay Kit (Pierce). NuPAGE LDS sample buffer containing DTT (100 μmol/L) was added to the lysate before the samples were subjected to immunoblotting as previously described.

### Data analysis of The Cancer Genome Atlas and Molecular Taxonomy of Breast Cancer International Consortium cohorts

Gene-level somatic copy number and RNA-seq data for breast cancer [The Cancer Genome Atlas (TCGA) BRCA] were downloaded from the Genomic Data Commons (GDC). *FGFR1* copy-number estimates were obtained from SNP array data processed through the GDC pipeline. Gene expression data were obtained as STAR-aligned raw counts (unstranded) and normalized using variance-stabilizing transformation with the DESeq2 package (24). ER and HER2 status were inferred from extended histologic annotations provided by Thennavan and colleagues (27). Clinical endpoint data were obtained from TCGA Pan-Cancer Clinical Data Resource (TCGA-CDR; ref. 28). Disease-free survival (DFS) was defined using the disease-free interval (DFI); in the absence of a recorded relapse event, disease-specific survival (DSS) was used as a surrogate for recurrence. If neither a DFI nor a DSS event was recorded, patients were censored at the time of last follow-up. Patients with unknown relapse status and no recorded DSS event were excluded from the DFS analysis. Gene expression, somatic copy-number alteration (SCNA), and clinical data from the Molecular Taxonomy of Breast Cancer International Consortium (METABRIC) cohort were used for this analysis (29). Normalized gene expression data from the discovery (*n* = 997) and validation (*n* = 983) cohorts were merged. Probe-level expression data from the Illumina HumanHT-12 v3 microarray platform were collapsed to the gene level by mapping Illumina probe IDs to HUGO Gene Nomenclature Committee gene symbols using the illuminaHumanv3.db annotation package and averaging expression values across all probes corresponding to the same gene. Clinical annotations were obtained from the molecular dataset curated by Rueda and colleagues (30). SCNA data were processed using ASCAT v2.3 segmentation files. Only samples with matched expression, copy number, and clinical data were retained for downstream analysis. In METABRIC, DFS was defined using the earliest occurrence of locoregional (LR) or distant relapse (DR); in the absence of a recorded relapse event (i.e., LR = 0 and DR = 0), DSS was used as a surrogate for recurrence. If neither a relapse nor a DSS event was recorded, patients were censored at the time of last follow-up. Kaplan–Meier survival analyses were performed using the survminer R package (v0.4.9, RRID: SCR_021094), and Cox proportional hazards models were fitted using the survival R package (v3.7-0, RRID: SCR_021137). Univariable Cox models were summarized using the gtsummary package (v2.0.4, RRID: SCR_021319). For TCGA and METABRIC, *FGFR1* amplification was defined as an estimated copy number ≥6.

## Results

### FGFR1-overexpressing tamoxifen-resistant breast cancer cells exhibit increased dsDNA foci and IFN response

**Figure 1.**
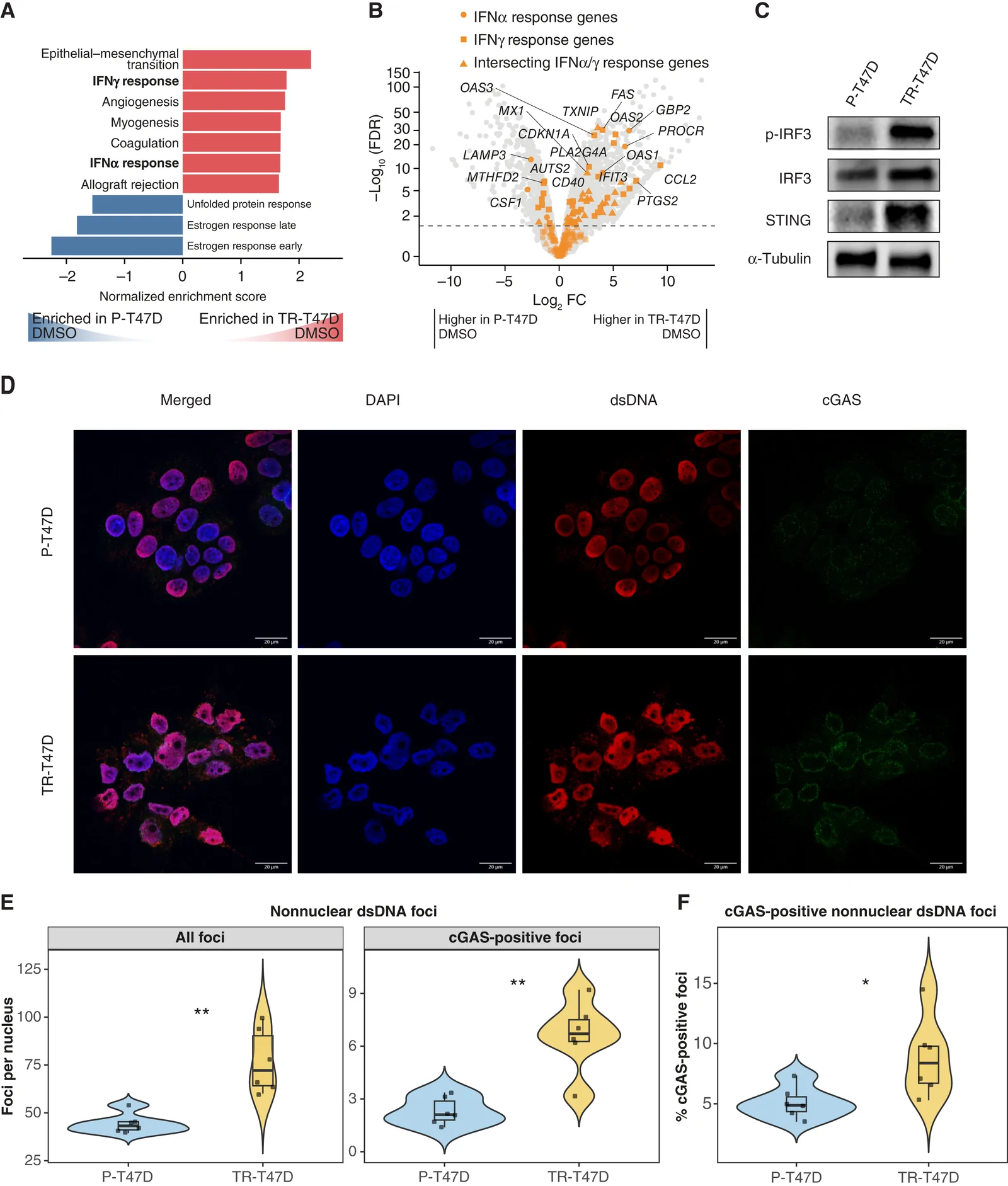
**A,** GSEA of hallmark pathways comparing TR-T47D vs. P-T47D. **B,** Differentially expressed genes in TR-T47D vs. P-T47D. The dashed horizontal line represents the threshold for statistical significance (FDR < 0.05). **C,** Immunoblots of p-IRF3, total IRF3, and STING in P-T47D and TR-T47D cells. α-Tubulin, loading control. **D,** Immunofluorescence images of DAPI (blue), dsDNA (red), and cGAS (green) in P-T47D (top) and TR-T47D (bottom) cells. Scale bars, 20 μm. **E,** Quantification of nonnuclear dsDNA foci per nucleus in P-T47D and TR-T47D cell colonies, shown for all foci (left) and cGAS-positive foci (right). *N* = 6 images per cell line. **F,** Percentage of nonnuclear dsDNA foci classified as cGAS positive per image in P-T47D and TR-T47D cells. *N* = 6 images per cell line. Violin plots show the data distribution with overlaid boxplots displaying the median and interquartile range (IQR); whiskers extend to the most extreme data point within 1.5 × IQR of the box edges. *P* values are indicated by asterisks (*, *P* < 0.05; **, *P* < 0.01).

To investigate the role of FGFR1 in endocrine resistance, we examined TR-T47D cells characterized by FGFR1 upregulation at both the protein and mRNA levels (Supplementary Fig. S1A and S1B). RNA-seq followed by GSEA comparing TR-T47D and P-T47D cells revealed significant enrichment of the MSigDB hallmark IFNα and IFNγ response gene sets (Fig. 1A), as well as higher expression of IFN response genes in TR-T47D cells (Fig. 1B). Although the IFNα and IFNγ hallmark gene sets are defined independently, TR-T47D cells exhibited a broad upregulation of genes across both signatures, hereafter collectively referred to as IFN response genes. Tamoxifen has previously been linked to DNA double-strand breaks (DSB) and replication stress (4, 7), raising the possibility that cytosolic dsDNA accumulation and cGAS–STING activation contribute to the elevated IFN response observed in TR-T47D cells. We therefore explored markers of cGAS–STING pathway activation in these cells. Immunoblotting revealed elevated total STING protein and increased p-IRF3 in TR-T47D cells relative to P-T47D cells, with equivalent total IRF3 across conditions (Fig. 1C). Consistent with elevated STING expression at the protein level, STING mRNA was also significantly higher in TR-T47D cells (Supplementary Fig. S1C). Phosphorylated STING and phosphorylated TBK1 were not detected under these conditions. To assess whether this activation is associated with cytosolic nucleic acid sensing, we performed immunocytochemistry for cGAS and dsDNA (Fig. 1D). Quantification of nonnuclear dsDNA foci demonstrated a significant increase in the total number of dsDNA foci in TR-T47D cells [Fig. 1E (left)]. Moreover, the number of cGAS-positive foci was also significantly elevated in TR-T47D cells [Fig. 1E (right)], and TR-T47D cells exhibited a significantly higher proportion of cGAS-positive nonnuclear dsDNA foci relative to cGAS-negative foci compared with P-T47D cells (Fig. 1F). In addition, TR-T47D cells displayed significantly higher nuclear cGAS intensity compared with P-T47D cells [Fig. 1D; Supplementary Fig. S1D (left)], with no significant difference in nonnuclear cGAS levels [Supplementary Fig. S1D (right)]. Collectively, these data indicate that TR-T47D cells accumulate cytosolic dsDNA associated with cGAS engagement and constitutive IRF3 activation, consistent with cGAS–STING–driven IFN response gene expression.

### FGFR1 inhibition restores tamoxifen sensitivity and potentiates IFN response gene expression in tamoxifen-resistant cells

**Figure 2.**
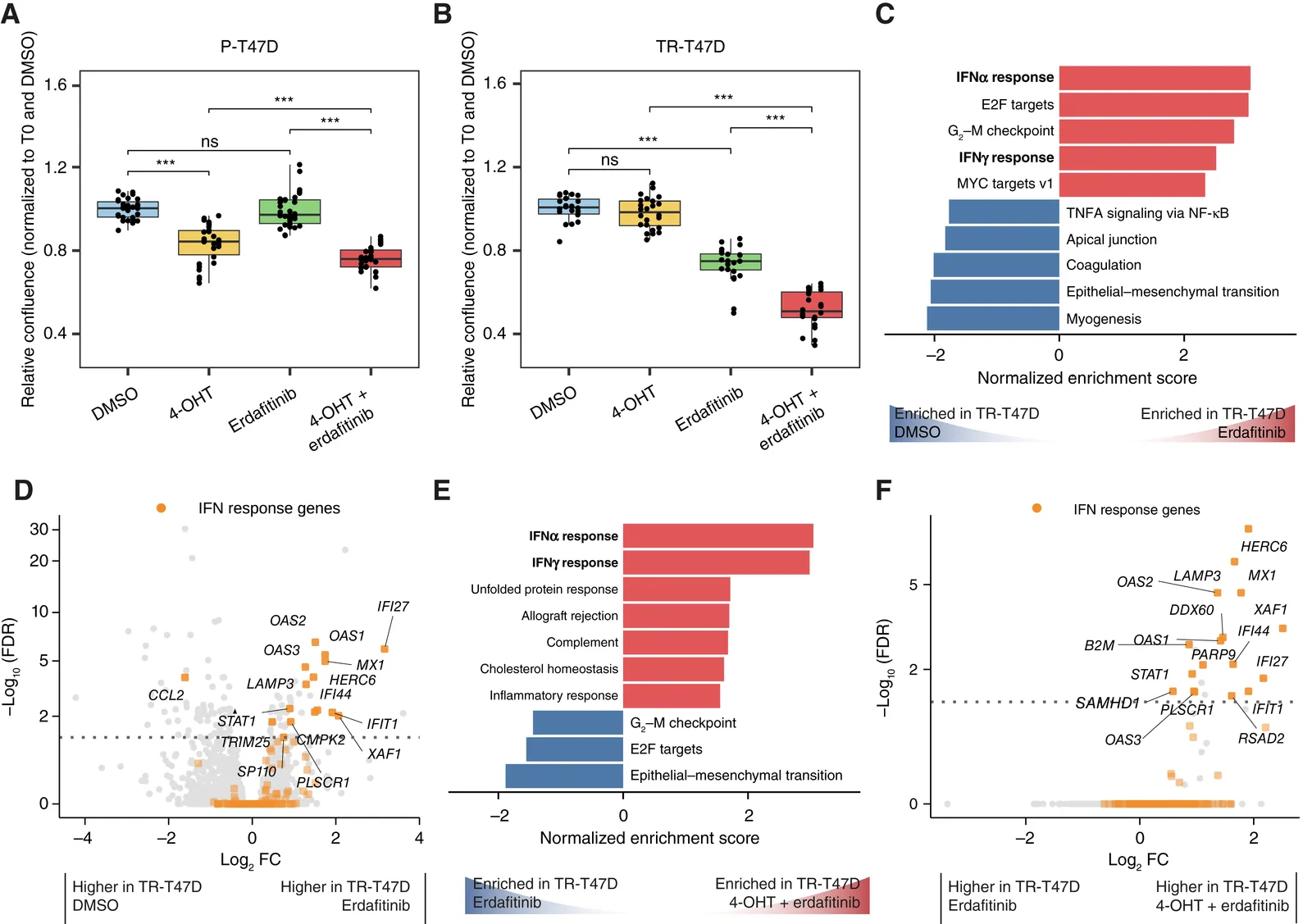
**A** and **B,** Relative cell confluence at 96 hours in (**A**) P-T47D and (**B**) TR-T47D cells treated with DMSO, 4-OHT, erdafitinib, or their combination. Boxplots show the median and IQR with whiskers extending to the most extreme data point within 1.5 × IQR of the box edges. Each point represents a technical replicate nested within a biological replicate (*n* = 5 for P-T47D; *n* = 4 for TR-T47D); points are horizontally offset to reflect their biological replicate grouping. Adjusted *P* values (Tukey) indicated by asterisks (ns, nonsignificant; ***, *P* < 0.001). **C,** GSEA of hallmark pathways comparing TR-T47D cells treated with erdafitinib vs. DMSO. Bars represent normalized enrichment scores for the top 10 most significantly enriched gene sets (FDR < 0.05). **D,** Differentially expressed genes in erdafitinib- vs. DMSO-treated TR-T47D cells. The dashed horizontal line represents the threshold for statistical significance (FDR < 0.05). **E,** GSEA of hallmark pathways comparing TR-T47D cells treated with erdafitinib + 4-OHT vs. erdafitinib. Bars represent normalized enrichment scores for the top 10 most significantly enriched gene sets (FDR < 0.05). **F,** Differentially expressed genes in erdafitinib + 4-OHT– vs. erdafitinib-treated TR-T47D cells. The dashed horizontal line represents the threshold for statistical significance (FDR < 0.05).

To investigate the functional role of FGFR1 in endocrine resistance, we performed proliferation assays in P-T47D and TR-T47D cells following treatment with 4-OHT and the selective FGFR inhibitor erdafitinib. P-T47D cells were sensitive to 4-OHT but did not respond to erdafitinib monotherapy (Fig. 2A; Supplementary Fig. S2A and S2B). TR-T47D cells were insensitive to 4-OHT yet highly sensitive to erdafitinib, and the combination of 4-OHT and erdafitinib significantly suppressed growth in both cell lines, demonstrating that FGFR signaling contributes to tamoxifen resistance and that its inhibition can restore 4-OHT sensitivity (Fig. 2B; Supplementary Fig. S2C and S2D). To characterize the transcriptional effects of FGFR1 inhibition, we performed RNA-seq of TR-T47D cells treated with DMSO, erdafitinib, or erdafitinib combined with 4-OHT. GSEA revealed that erdafitinib alone significantly enriched IFN response genes relative to DMSO-treated TR-T47D cells (Fig. 2C), implicating FGFR1 as a suppressor of IFN signaling in these cells. Differential expression analysis confirmed upregulation of IFN response genes following erdafitinib treatment (Fig. 2D). Although 4-OHT treatment alone exerted minimal transcriptional effects in TR-T47D cells (Supplementary Fig. S2E), its combination with erdafitinib resulted in a marked increase in IFN response genes compared with erdafitinib monotherapy (Fig. 2E and F), several of which—*XAF1*, *STAT1*, *IFI27*, and *SAMHD1*—are associated with IFN-induced apoptosis (31–34). These findings demonstrate that erdafitinib restores the ability of 4-OHT to induce IFN response gene expression. To validate the specific effect of FGFR1 in this context, we generated TR-T47D cells with stable shRNA-mediated *FGFR1* knockdown (TR-T47D^shFGFR1^; Supplementary Fig. S2F) and assessed 4-OHT sensitivity by cell viability assay. *FGFR1* knockdown restored 4-OHT sensitivity (Supplementary Fig. S2G), consistent with the observed effect of erdafitinib. Additionally, RNA-seq followed by GSEA and differential expression analysis of TR-T47D^shFGFR1^ versus TR-T47D^shCTRL^ cells identified significant enrichment and differential expression of IFN response genes (Supplementary Fig. S2H and S2I). Taken together, pharmacologic and genetic suppression of FGFR1 derepresses IFN response gene expression and potentiates 4-OHT–induced IFN response gene expression in tamoxifen-resistant cells.

### FGFR1 suppresses STING-mediated IFN response in tamoxifen-resistant cells

**Figure 3.**
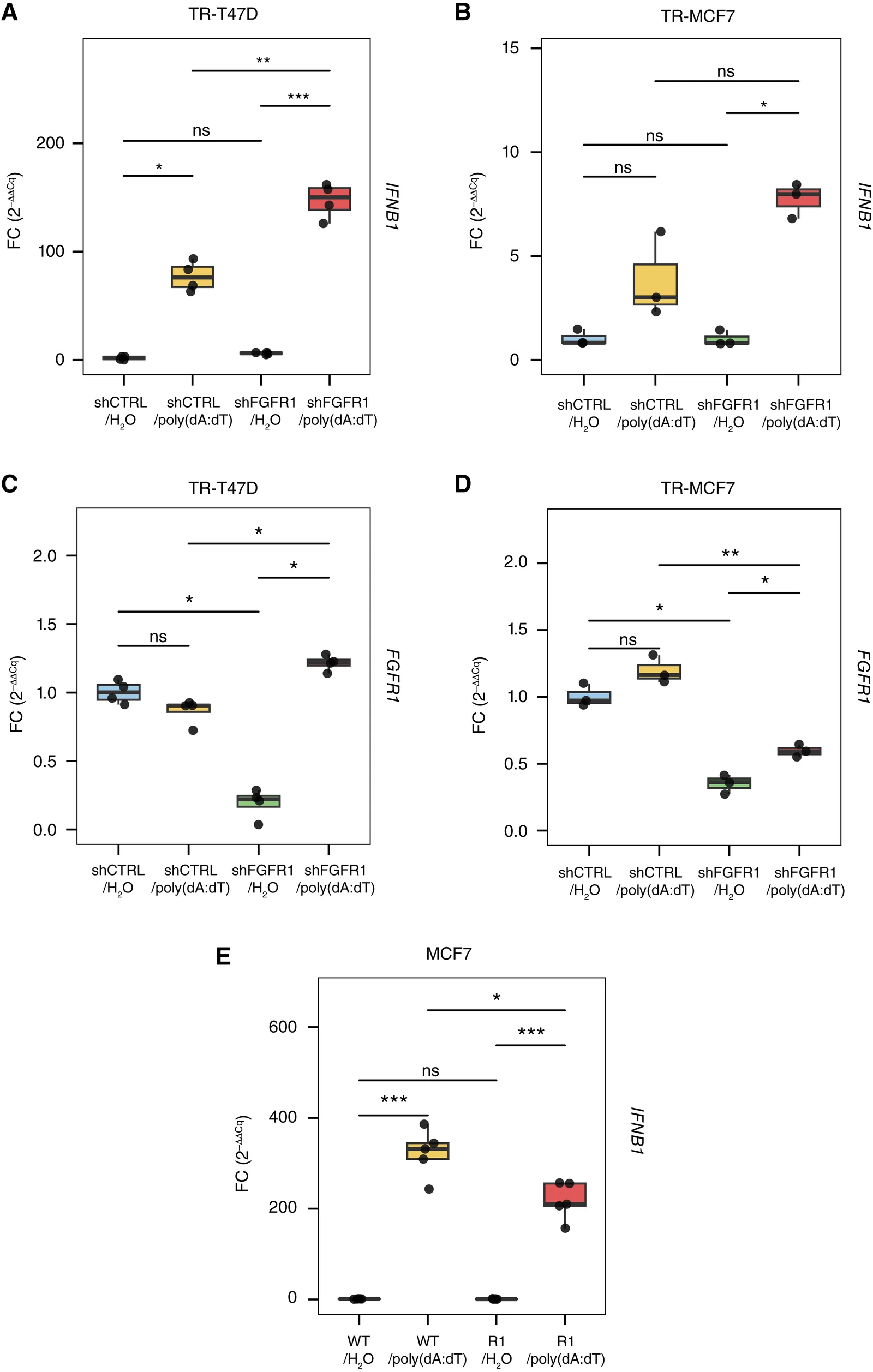
Relative expression of *IFNB1* (**A** and **B**) and *FGFR1* (**C** and **D**) in TR-T47D^shCTRL^/TR-T47D^shFGFR1^ cells and TR-MCF7^shCTRL^/TR-MCF7^shFGFR1^ cells, respectively, treated with either H_2_O or poly(dA:dT), measured by RT-qPCR normalized to *GAPDH* and *ACTB*. FC was calculated relative to the geometric mean of ΔCq of the shCTRL/H_2_O condition. **E,** Relative expression of *IFNB1* in WT-MCF7 and R1-MCF7 cells treated with H_2_O or poly(dA:dT), measured by RT-qPCR and normalized to *HPRT1*. FC was calculated relative to the geometric mean of ΔCq of the WT-MCF7/H_2_O condition. Boxplots show the median and IQR with whiskers extending to the most extreme data point within 1.5 × IQR of the box edges; individual points represent biological replicates. Adjusted *P* values (Benjamini–Hochberg) are indicated by asterisks (ns, nonsignificant; *, *P* < 0.05; **, *P* < 0.01; ***, *P* < 0.001).

Having established that FGFR1 inhibition induces IFN response gene expression in TR-T47D cells, we next sought to determine if FGFR1 directly modulates STING-mediated IFN response. We stimulated *FGFR1* knockdown and controls of two tamoxifen resistant cell lines (TR-T47D^shFGFR1^/TR-T47D^shCTRL^ and TR-MCF7^shFGFR1^/TR-MCF7^shCTRL^) with poly(dA:dT), a synthetic dsDNA analogue that activates STING via cGAS-dependent sensing. TR-MCF7 is characterized by elevated *FGFR1* mRNA and corresponding FGFR1 protein levels (Supplementary Fig. S3A and S3B) and significantly elevated *STING1* mRNA expression and STING1 protein abundance relative to WT-MCF7 (Supplementary Fig. S3C and S3D). Strikingly, poly(dA:dT)-induced expression of *IFNB1* was significantly enhanced in TR-T47D^shFGFR1^ cells compared with TR-T47D^shCTRL^ (Fig. 3A), further supporting the role of FGFR1 as a negative regulator of STING-mediated IFN response in tamoxifen-resistant breast cancer cells. The same trend was observed in TR-MCF7 cells, although the increase in poly(dA:dT)-induced *IFNB1* expression between TR-MCF7^shFGFR1^ and TR-MCF7^shCTRL^ cells did not reach statistical significance (Fig. 3B). Poly(dA:dT)-induced *IFNA2* expression was not affected by *FGFR1* knockdown in either TR-T47D or TR-MCF7 cells (Supplementary Fig. S3E and S3F). We also measured the expression of *STING1* across the same treatment conditions. Although poly(dA:dT)-induced *STING1* expression was not significantly increased in TR-T47D^shFGFR1^ compared with TR-T47D^shCTRL^ cells (Supplementary Fig. S3G), *STING1* expression was significantly increased in TR-MCF7^shFGFR1^ compared with TR-MCF7^shCTRL^ (Supplementary Fig. S3H). We measured *FGFR1* expression upon poly(dA:dT) treatment. *FGFR1* expression was not affected by poly(dA:dT) treatment in TR-T47D^shCTRL^ and TR-MCF7^shCTRL^ cells (Fig. 3C and D). Strikingly, *FGFR1* expression was either partially or completely restored by poly(dA:dT) treatment in TR-T47D^shFGFR1^ and MCF7^shFGFR1^ cells, respectively. To confirm that *FGFR1* overexpression is sufficient to suppress STING-mediated IFN response, we treated R1-MCF7 with poly(dA:dT). Although poly(dA:dT) robustly induced *IFNB1* expression in WT-MCF7 cells, this response was significantly attenuated in R1-MCF7 cells (Fig. 3E). Together, these findings support a model in which FGFR1 suppresses STING-mediated IFN response and is reciprocally induced as a negative feedback regulator.

### FGFR1 inhibition enhances tamoxifen-induced IFN response *in vivo*

**Figure 4.**
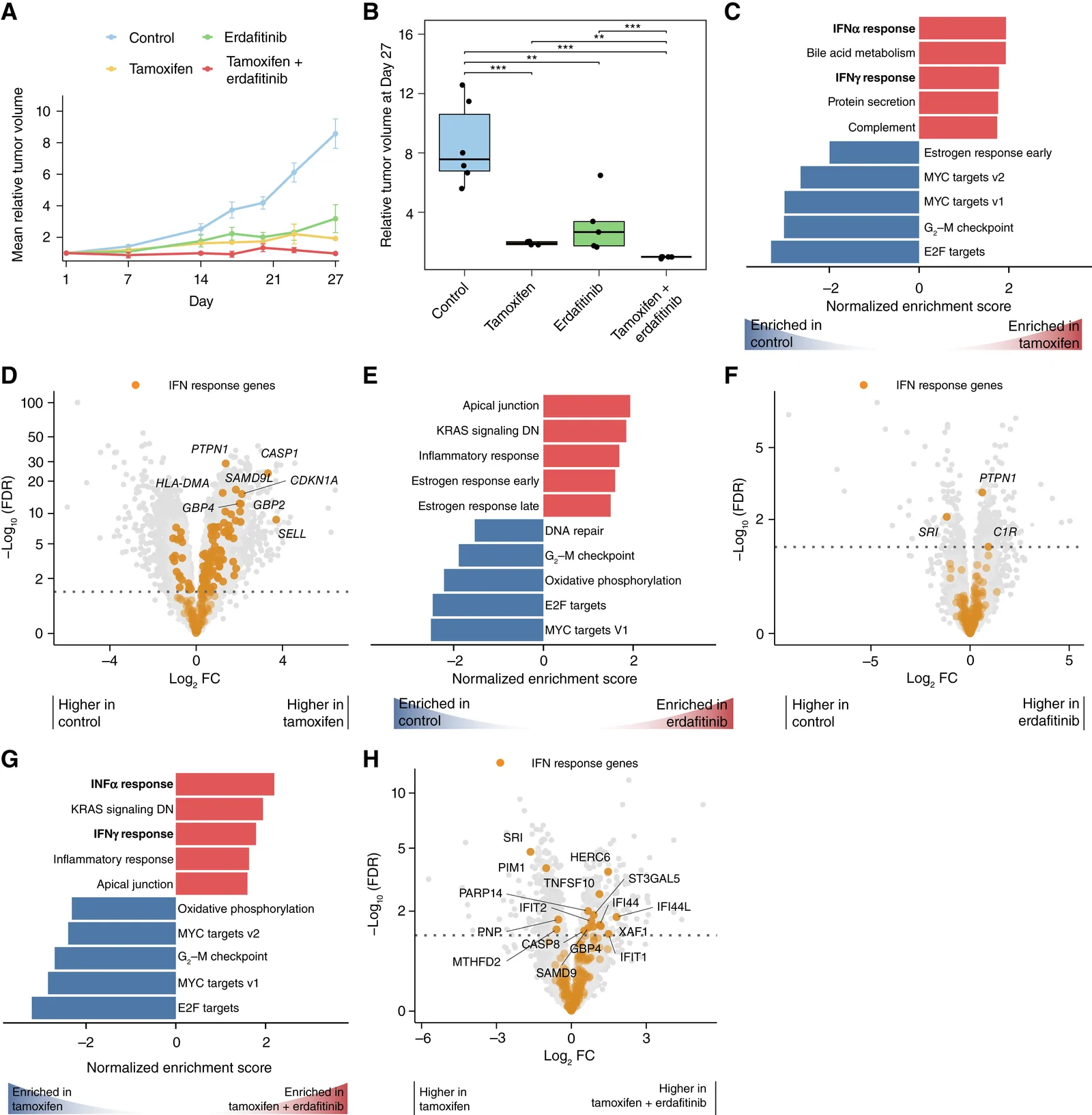
**A,** Relative tumor growth of MAS98.06 breast cancer PDX tumors (control, tamoxifen, erdafitinib, or tamoxifen + erdafitinib treated), normalized to the initial volume at treatment start (day 1). Error bars indicate the standard error of the mean (SEM) at each time point. Only tumors with initial volume between 50 and 300 mm^3^ were included in the analysis. **B,** Relative tumor volumes at day 27 for the tumors included in **A**. Each point represents a single tumor. Boxplots show the median and IQR with whiskers extending to the most extreme data point within 1.5 × IQR of the box edges. Adjusted *P* values (Benjamini–Hochberg) are indicated by asterisks (**, *P* < 0.01; ***, *P* < 0.001). **C,** GSEA of hallmark pathways in tamoxifen-treated vs. control tumors. Bars represent normalized enrichment scores for the five most significantly enriched gene sets in each direction (FDR < 0.05). **D,** Differentially expressed genes in tamoxifen-treated vs. control tumors. The dashed horizontal line indicates the threshold for statistical significance (FDR < 0.05). **E,** GSEA of hallmark pathways in erdafitinib-treated vs. control tumors. Bars represent normalized enrichment scores for the five most significantly enriched gene sets in each direction (FDR < 0.05). **F,** Differentially expressed genes in erdafitinib-treated vs. control tumors. The dashed horizontal line indicates the threshold for statistical significance (FDR < 0.05). **G,** GSEA of hallmark pathways in tamoxifen + erdafitinib–treated vs. tamoxifen-treated tumors. Bars represent normalized enrichment scores for the five most significantly enriched gene sets in each direction (FDR < 0.05). **H,** Differentially expressed genes in tamoxifen + erdafitinib–treated vs. tamoxifen-treated tumors. The dashed horizontal line indicates the threshold for statistical significance (FDR < 0.05).

To determine whether induction of IFN response gene expression represents a direct transcriptional consequence of tamoxifen rather than an adaptation acquired during the development of resistance, we utilized a treatment-naïve, *FGFR1*-amplified PDX model (Supplementary Fig. S4A and S4B). This allowed us to interrogate the transcriptional effects of tamoxifen and FGFR1 inhibition prior to any resistance-associated transcriptional remodeling. Single-agent treatment with erdafitinib and tamoxifen both resulted in significant tumor growth inhibition (Fig. 4A and B; Supplementary Fig. S4C and S4D). Consistent with our findings *in vitro*, combined treatment with tamoxifen and erdafitinib resulted in significantly greater tumor growth inhibition compared with either agent alone (Fig. 4B). RNA-seq followed by GSEA revealed a robust enrichment of IFN response genes in tamoxifen-treated tumors compared with controls (Fig. 4C and D). Additionally, tamoxifen treatment induced expression of *FGFR1* and *STING1* (Supplementary Fig. S4E). Notably, although erdafitinib alone effectively inhibited tumor growth, GSEA did not show enrichment of IFN response genes (Fig. 4E and F). However, when added to tamoxifen, erdafitinib enhanced tamoxifen-induced enrichment and differential expression of IFN response genes (Fig. 4G and H). Together, these findings suggest that induction of IFN response genes and *FGFR1* is a direct transcriptional consequence of tamoxifen treatment and that FGFR1 activity attenuates this effect.

### *STING1* expression influences the prognostic impact of *FGFR1* amplification in ER^+^/HER2^−^ breast cancer

**Figure 5.**
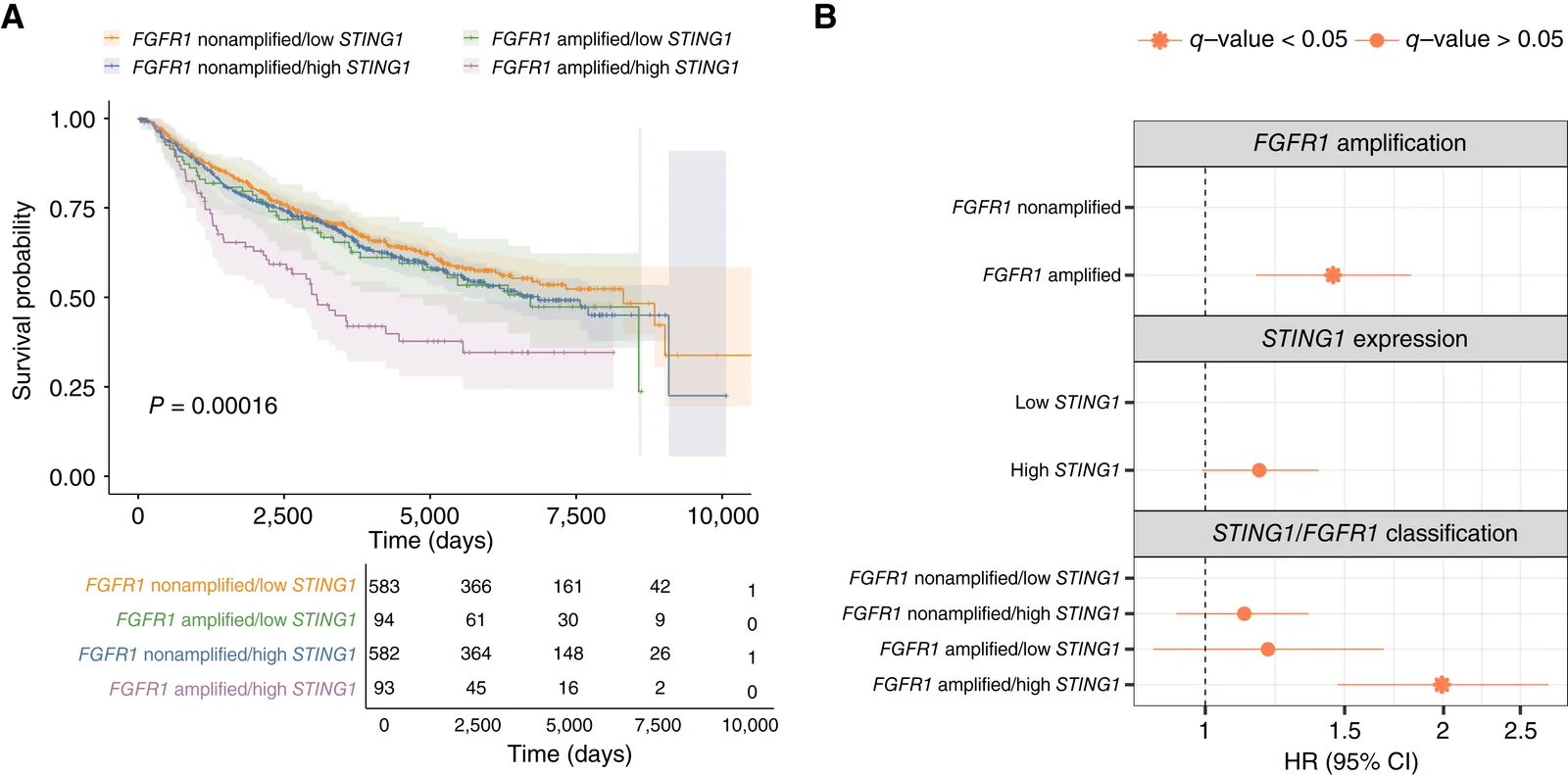
**A,** DFS stratified by combined *FGFR1* amplification and *STING1* expression status in ER^+^/HER2^−^ breast tumors from the METABRIC cohort. *FGFR1* amplification was defined as copy number ≥6. *STING1* expression was dichotomized at the median into “high *STING1*” and “low *STING1*.” Log-rank *P* values and 95% confidence intervals (CI). **B,** Univariate Cox regression analysis of DFS in ER^+^/HER2^−^ breast tumors from the METABRIC cohort, stratified by *FGFR1* amplification and *STING1* expression. HRs and 95% CIs. *FGFR1* amplification was defined as copy number ≥6, and *STING1* expression was dichotomized at the median into “high *STING1*” and “low *STING1*.” The four resulting subgroups are the same as those shown in **A**. HRs are displayed on a log_10_ scale, with the reference group set as “*FGFR1* nonamplified/low *STING1*.” Point shapes indicate statistical significance following FDR correction (q value < 0.05).

To investigate whether the suppressive impact of FGFR1 on STING-mediated IFN response is reflected in clinical outcomes, we examined the prognostic effect of *FGFR1* amplification stratified by *STING1* expression in ER^+^/HER2^−^ breast cancer. In the METABRIC cohort, Kaplan–Meier analysis comparing tumors with and without *FGFR1* amplification and high/low *STING1* expression showed significantly different DFS, with *FGFR1*^Amplified^/*STING1*^High^ tumors exhibiting the worst outcome (Fig. 5A). Interestingly, among tumors with low *STING1* expression, the difference in DFS between *FGFR1*-amplified and nonamplified tumors was much smaller than that among tumors with high *STING1* expression. Univariate Cox proportional hazards analysis confirmed that *FGFR1* amplification alone was significantly associated with worse DFS, whereas *STING1* expression alone was not (Fig. 5B). Among the four combined subgroups, only *FGFR1*^Amplified^/*STING1*^High^ tumors reached statistical significance, carrying the highest hazard ratio (HR) of any subgroup (Fig. 5B). A similar pattern was observed in TCGA-BRCA cohort, although statistical significance was not reached, likely reflecting reduced cohort size (Supplementary Fig. S5A and S5B). Together, these clinical data support the model established *in vitro* and *in vivo*, whereby *FGFR1* amplification confers its adverse effect specifically in tumors with high *STING1* expression, consistent with FGFR1 acting as a suppressor of STING-driven IFN signaling in a subset of ER^+^ breast cancer.

## Discussion

The role of FGFR1 in endocrine-resistant breast cancer has been extensively investigated, yet clinical evaluation of FGFR-targeted therapies has consistently demonstrated limited benefit (35, 36). This may in part reflect inadequate patient stratification through an overreliance on *FGFR1* amplification as a proxy for functional oncogenic dependency. Defining the precise molecular conditions under which FGFR1 drives resistance is therefore a prerequisite for identifying the patients most likely to benefit from its inhibition. Here, we present evidence that FGFR1 operates as a suppressor of IFN response in tamoxifen-resistant breast cancer and explore the implications of this mechanism for biomarker identification and patient stratification.

Tamoxifen is an established genotoxic agent, capable of generating DNA damage through multiple mechanisms including formation of reactive metabolites and DSBs (4). Tamoxifen treatment has been shown to induce the accumulation of cytoplasmic DNA in breast cancer cells, proposed to engage innate immune DNA sensors and drive ISG expression (7). Consistent with this precedent, we observe an increased number of nonnuclear dsDNA foci in tamoxifen-resistant cells, a significantly greater proportion of which are cGAS positive relative to parental controls cells. Whether these foci represent cytosolic DNA derived from DNA damage, micronuclei, or other sources of chromatin displacement cannot be determined from dsDNA immunofluorescence alone. However, their co-occurrence with elevated total *STING* mRNA and STING protein, constitutive IRF3 phosphorylation, and broad transcriptional upregulation of IFN response genes point toward sustained cGAS–STING signaling as a feature of the tamoxifen-resistant state. Given that FGF–FGFR signaling has been documented to directly antagonize IFN signaling (8), this raises the question of whether FGFR1 is upregulated as a negative regulator of the IFN response, thereby allowing tamoxifen-resistant cells to tolerate and persist under chronic IFN signaling. Erdafitinib treatment and *FGFR1* knockdown both induced IFN response gene expression in TR-T47D cells, consistent with evidence that FGFR signaling suppresses ISG expression in murine antiviral defense. FGFR1 suppression further potentiated poly(dA:dT)-driven *IFNB1* induction, implicating FGFR1 kinase activity in restraining STING-mediated *IFNB1* transcription. Reciprocally, poly(dA:dT) treatment increased *FGFR1* expression, consistent with evidence that type I IFN signaling can transcriptionally induce *FGFR1* expression (37), suggesting a negative feedback loop in which IFNs induce expression of its own negative regulator.

The concurrent elevation of STING and FGFR1 in tamoxifen-resistant cells is consistent with an IFN–FGFR1 feedback mechanism. STING’s capacity for feedforward amplification of its own expression (38) would likely drive sustained type I IFN production under chronic cytosolic DNA accumulation, creating selective pressure for negative regulators capable of attenuating this output. We propose that chronic exposure to tamoxifen imposes a selection pressure for cells upregulating FGFR1 as an adaptive mechanism to restrain STING feedforward signaling, permitting tolerance of cytosolic DNA accumulation. Consistent with this model, tamoxifen induced expression of IFN response genes in TR-T47D cells only in the context of concurrent FGFR1 inhibition. This indicates that FGFR1 kinase activity suppresses tamoxifen-driven IFN expression in the resistant state and that this suppression can be derepressed to restore the effects of tamoxifen.

This result was further validated in an *FGFR1*-amplified PDX model prior to any resistance-associated transcriptional remodeling. Tamoxifen treatment induced IFN response gene enrichment and led to elevated *STING1* and *FGFR1* expression *in vivo*, confirming that this pattern is observed by tamoxifen treatment itself and is not contingent on the acquired resistant state. Erdafitinib monotherapy suppressed tumor growth without enriching IFN response genes, yet combination with tamoxifen augmented IFN response gene expression beyond that of tamoxifen treatment alone.

Despite evidence from preclinical models implicating FGFR1 as a driver of endocrine resistance in breast cancer, translating this biology into therapeutic benefit for patients has proved challenging. In the *RADICAL* trial, combining AZD4547 with endocrine therapy yielded only modest clinical benefit, with an objective response rate of approximately 10% and frequent toxicities limiting treatment duration (35). Similarly, in the *NCI-MATCH* trial, erdafitinib failed to produce any confirmed objective responses in patients with *FGFR*-amplified tumors, highlighting the limited efficacy of FGFR-targeted monotherapy in this context (36). A challenge across these studies is the absence of predictive biomarkers that can reliably identify tumors truly dependent on FGFR1 signaling, reflecting our limited understanding of the molecular conditions under which FGFR1 acts as an oncogenic driver.

The survival analyses presented here suggest that additional biomarkers such as *STING1* may be codeterminants of FGFR1 dependency. In the METABRIC cohort, the prognostic impact of *FGFR1* amplification was contingent on tumor *STING1* expression. This stratification pattern is consistent with *STING1* expression being a potential biomarker of FGFR1 dependency. If FGFR1’s contribution to tumor progressions operates primarily through suppression of STING-mediated IFN response, its amplification would be expected to confer a selective advantage in tumors in which such signaling is present. Although *STING1* mRNA expression provides an accessible identification of pathway activity, refining this association will require broader functional characterization of cGAS–STING activity within the tumor. Taken together, these data suggest that *FGFR1* amplification and STING may be used as prognostic variables to define a subset of tumors that are most likely to be FGFR1 dependent, with direct implications for stratification in trials of FGFR-targeted therapy.

The precise molecular mechanism through which FGFR1 suppresses cGAS–STING–driven IFN signaling remains unresolved, and the primary cell line evidence from our work is largely derived from MCF7- and T47D-based resistant models, warranting validation across broader ER^+^ breast cancer contexts. Although STING has been proposed as a tumor suppressor in some contexts, chronic activation has also been linked to signal rewiring away from IFN I toward noncanonical NF-κB and therapeutic resistance (39, 40). Whether FGFR1 similarly redirects STING output toward this branch, rather than simply suppressing it, remains to be tested. Furthermore, *STING1* expression as assessed in METABRIC represents a transcriptional proxy rather than a direct measure of pathway activity, and the relative contribution of genomic *FGFR1* amplification versus IFN-driven transcriptional induction to FGFR1 upregulation in the clinical setting remains unclear. Future work should aim to resolve the mechanistic basis of FGFR1-mediated innate immune suppression and evaluate whether functional cGAS–STING status can be assessed in a biopsy-compatible format to prospectively stratify patients for evaluation of FGFR-targeted therapy.

## Supplementary Material

Supplementary Figure 1 A) Western blot shows FGFR1 protein levels in P-T47D and TR-T47D cells. GAPDH as loading control. (B–C) Relative expression of *FGFR1* and *STING1* respectively, in P-T47D and TR-T47D cells, measured by RT-qPCR and normalized to *ACTB* and *RPLP1*. Fold change was calculated relative to the geometric mean of ΔCq of the P-T47D condition. Boxplots show the median and IQR with whiskers extending to the most extreme data point within 1.5 × IQR of the box edges; individual points represent biological replicates. D) Mean cGAS immunofluorescence intensity in P-T47D and TR-T47D cells in nuclear (left; each point = one nucleus) and non-nuclear (right; each point = one image) compartments.

Supplementary Figure 2 (A–B) Growth curves of P-T47D cells treated with DMSO, 4-OHT, erdafitinib, or the combination of 4-OHT and erdafitinib. (A) Mean relative confluence ± standard error (SE), normalized to the starting timepoint (T = 0), shown separately for five biological replicates. (B) Individual growth curves for all technical replicates within each biological replicate shown in panel A, illustrating intra-replicate variability. (C–D) Growth curves of TR-T47D cells treated DMSO, 4-OHT, erdafitinib, or the combination of 4-OHT and erdafitinib. (C) Mean relative confluence ± SE, normalized to the starting timepoint (T = 0), shown separately for four biological replicates. (D) Individual growth curves for all technical replicates within each biological replicate shown in panel C, illustrating intra-replicate variability. E) Differentially expressed genes in TR-T47D cells treated with 4-OHT versus DMSO. The dashed horizontal line indicates the threshold for statistical significance (FDR < 0.05). F) Western blot shows FGFR1 protein levels in TR-T47D^shCTRL^ and TR-T47D^shFGFR1^ cells. GAPDH as loading control. G) Cell viability was measured using the MTS assay across a range of 4-OHT concentrations in P-T47D cells, TR-T47D^shFGFR1^ and TR-T47D^shCTRL^ cells. Data are shown as mean viability ± standard error (SE) relative to DMSO-treated controls. The x-axis represents the log10-transformed 4-OHT concentration (µM); the dashed line indicates 100% viability. H) GSEA of Hallmark pathways in TR-T47D^shFGFR1^ versus TR-T47D^shCTRL^ cells. Bars represent normalized enrichment scores for the five most significantly enriched gene sets in each direction (FDR < 0.05). I) Differentially expressed genes in TR-T47D^shFGFR1^ cells versus TR-T47D^shCTRL^.

Supplementary Figure 3 A) Western blot shows FGFR1 protein levels in WT-MCF7 and TR-MCF7 cells. GAPDH was as loading control. B) Relative expression of *FGFR1* in WT-MCF7 and TR-MCF7 cells, measured by RT–qPCR and normalized to *ACTB* and *RPLP1*. Fold change was calculated relative to the geometric mean of ΔCq of the MCF7-WT condition. C) Western blot shows STING protein levels in WT-MCF7 and TR-MCF7. Total protein as loading control. D) Relative expression of *STING1* in WT-MCF7 and TR-MCF7 cells measured by RT–qPCR and normalized to *ACTB* and *RPLP1*. Fold change was calculated relative to the geometric mean of ΔCq of the MCF7-WT condition. E–H) Relative expression of *IFNA2* (E-F) and ^STING1^ (G–H) in TR-T47D^shCTRL^/TR-T47D^shFGFR1^ cells and TR-MCF7^shCTRL^/TR-MCF7^shFGFR1^ cells respectively, treated with either H_2_O or poly(dA:dT), measured by RT–qPCR and normalized to *GAPDH* and *RPLP1*. Fold change was calculated relative to the geometric mean of ΔCq of the shCTRL/H_2_O condition. I) Relative expression of *FGFR1* in WT-MCF7 and R1-MCF7 cells, measured by RT-qPCR and normalized to *HPRT1*.

Supplementary Figure 4 A) Western blot shows FGFR1 protein levels in two luminal breast cancer PDX models MAS98.06 (*FGFR1* amplified) and HBCx34 (*FGFR1* nonamplified). β-actin as loading control. B) Array-based comparative genomic hybridization (aCGH) log_2_-ratio profile of chromosome 8 in MAS98.06. Each point represents an individual probe on a CGH microarray. Red points indicate probes mapping to the *FGFR1* locus (chr8: 38.17–38.58 Mb), highlighting a focal amplification. The top panel displays the full chromosome 8 profile, while the lower panel shows a zoomed-in view of the genomic region surrounding the *FGFR1* gene. The y-axis represents log_2_(test/reference) signal intensity, where values above zero indicate copy number gain and values below zero indicate loss. The x-axis indicates relative position along chromosome 8. C) Individual tumor growth of MAS98.06 PDX (control, tamoxifen, erdafitinib, or the combination tamoxifen + erdafitinib). Tumor volumes were measured at multiple time points, and each line represents a single tumor. Only tumors with initial volume between 50 and 300 mm³ were included in the analysis. D) Mean tumor volume for the MAS98.06 tumors shown in panel C. Lines represent the average tumor volume for each treatment group, and error bars indicate the standard error of the mean (SEM) at each time point. E) Differentially expressed genes in tamoxifen versus control treated MAS98.06 tumors. *FGFR1* and *STING1* are indicated.

Supplementary Figure 5 A) Disease-free survival (DFS) stratified by combined *FGFR1* amplification and *STING1* expression status in ER+/HER2– breast tumors from the TCGA-BRCA cohort. *FGFR1* amplification was defined as copy number ≥ 6. *STING1* expression was dichotomized at the median into “high *STING1*” and “low *STING1*”. Curves are truncated at 4,000 days due to the low number of patients at risk beyond this point. Log-rank P-values and 95% confidence intervals are shown. B) Univariate Cox regression analysis of DFS in ER+/HER2– breast tumors from the TCGA-BRCA cohort, stratified by *FGFR1* amplification and *STING1* expression. Hazard ratios (HRs) and 95% confidence intervals (CIs) are shown. *FGFR1* amplification was defined as copy number ≥ 6, and *STING1* expression was dichotomized at the median into “high *STING1*” and “low *STING1*” The four resulting subgroups are the same as those shown in panel A. Hazard ratios are displayed on a log10 scale, with the reference group set as “*FGFR1* nonamplified/low *STING1*”.

## Data Availability

RNA-seq data are available from the following repositories: dataset 1 (GSE200029) in Gene Expression Omnibus; dataset 2 (E-MTAB-17491) and dataset 3 (E-MTAB-17490) in ArrayExpress. Data and codes are available from https://github.com/taomli/Future_FGFR1_STING. Other data generated in this study are available upon request to the corresponding author.

## References

[1] Howlader N , Altekruse SF, Li CI, Chen VW, Clarke CA, Ries LAG, . US incidence of breast cancer subtypes defined by joint hormone receptor and HER2 status. J Natl Cancer Inst 2014;106:dju055. 10.1093/jnci/dju055. PMC4580552 24777111

[2] Cao LB , Ruan ZL, Yang YL, Zhang NC, Gao C, Cai C, . Estrogen receptor α-mediated signaling inhibits type I interferon response to promote breast carcinogenesis. J Mol Cell Biol 2024;15:mjad047. 10.1093/jmcb/mjad047. PMC11066933 37442610

[3] Bowie ML , Dietze EC, Delrow J, Bean GR, Troch MM, Marjoram RJ, . Interferon-regulatory factor-1 is critical for tamoxifen-mediated apoptosis in human mammary epithelial cells. Oncogene 2004;23:8743–55. 10.1038/sj.onc.1208120. 15467738

[4] Wozniak K , Kolacinska A, Blasinska-Morawiec M, Morawiec-Bajda A, Morawiec Z, Zadrozny M, . The DNA-damaging potential of tamoxifen in breast cancer and normal cells. Arch Toxicol 2007;81:519–27. 10.1007/s00204-007-0188-3. 17593413

[5] Schild-Hay LJ , Leil TA, Divi RL, Olivero OA, Weston A, Poirier MC. Tamoxifen induces expression of immune response–related genes in cultured normal human mammary epithelial cells. Cancer Res 2009;69:1150–5. 10.1158/0008-5472.CAN-08-2806. PMC2633418 19155303

[6] Schneider WM , Chevillotte MD, Rice CM. Interferon-stimulated genes: a complex web of host defenses. Annu Rev Immunol 2014;32:513–45. 10.1146/annurev-immunol-032713-120231. PMC4313732 24555472

[7] Post AEM , Smid M, Nagelkerke A, Martens JWM, Bussink J, Sweep FCGJ, . Interferon-stimulated genes are involved in cross-resistance to radiotherapy in tamoxifen-resistant breast cancer. Clin Cancer Res 2018;24:3397–408. 10.1158/1078-0432.CCR-17-2551. 29661777

[8] Maddaluno L , Urwyler C, Rauschendorfer T, Meyer M, Stefanova D, Spörri R, . Antagonism of interferon signaling by fibroblast growth factors promotes viral replication. EMBO Mol Med 2020;12:e11793. 10.15252/emmm.201911793. PMC7507082 32720440

[9] Wu S , Zhang Q, Zhang F, Meng F, Liu S, Zhou R, . HER2 recruits AKT1 to disrupt STING signalling and suppress antiviral defence and antitumour immunity. Nat Cell Biol 2019;21:1027–40. 10.1038/s41556-019-0352-z. 31332347

[10] Turner N , Pearson A, Sharpe R, Lambros M, Geyer F, Lopez-Garcia MA, . FGFR1 amplification drives endocrine therapy resistance and is a therapeutic target in breast cancer. Cancer Res 2010;70:2085–94. 10.1158/0008-5472.CAN-09-3746. PMC2832818 20179196

[11] Drago JZ , Formisano L, Juric D, Niemierko A, Servetto A, Wander SA, . FGFR1 amplification mediates endocrine resistance but retains TORC sensitivity in metastatic hormone receptor–positive (HR^+^) breast cancer. Clin Cancer Res 2019;25:6443–51. 10.1158/1078-0432.CCR-19-0138. PMC6825550 31371343

[12] Formisano L , Stauffer KM, Young CD, Bhola NE, Guerrero-Zotano AL, Jansen VM, . Association of FGFR1 with ERα maintains ligand-independent ER transcription and mediates resistance to estrogen deprivation in ER+ breast cancer. Clin Cancer Res 2017;23:6138–50. 10.1158/1078-0432.CCR-17-1232. PMC6681458 28751448

[13] Coombes RC , Badman PD, Lozano-Kuehne JP, Liu X, Macpherson IR, Zubairi I, . Author Correction: results of the phase IIa RADICAL trial of the FGFR inhibitor AZD4547 in endocrine resistant breast cancer. Nat Commun 2023;14:260. 10.1038/s41467-023-35969-4. PMC9845345 36650166

[14] Hui R , Pearson A, Cortes J, Campbell C, Poirot C, Azim HA Jr, . Lucitanib for the treatment of HR+/HER2− metastatic breast cancer: results from the multicohort phase II FINESSE study. Clin Cancer Res 2020;26:354–63. 10.1158/1078-0432.CCR-19-1164. 31619444

[15] Musolino A , Campone M, Neven P, Denduluri N, Barrios CH, Cortes J, . Phase II, randomized, placebo-controlled study of dovitinib in combination with fulvestrant in postmenopausal patients with HR+, HER2− breast cancer that had progressed during or after prior endocrine therapy. Breast Cancer Res 2017;19:18. 10.1186/s13058-017-0807-8. PMC5301372 28183331

[16] Liu X , Lai C, Wang K, Xing L, Yang P, Duan Q, . A functional role of fibroblast growth factor receptor 1 (FGFR1) in the suppression of influenza A virus replication. PLoS One 2015;10:e0124651. 10.1371/journal.pone.0124651. PMC4409105 25909503

[17] Ishikawa H , Barber GN. STING is an endoplasmic reticulum adaptor that facilitates innate immune signalling. Nature 2008;455:674–8. 10.1038/nature07317. PMC2804933 18724357

[18] Ishikawa H , Ma Z, Barber GN. STING regulates intracellular DNA-mediated, type I interferon-dependent innate immunity. Nature 2009;461:788–92. 10.1038/nature08476. PMC4664154 19776740

[19] Sun L , Wu J, Du F, Chen X, Chen ZJ. Cyclic GMP-AMP synthase is a cytosolic DNA sensor that activates the type I interferon pathway. Science 2013;339:786–91. 10.1126/science.1232458. PMC3863629 23258413

[20] Wu J , Sun L, Chen X, Du F, Shi H, Chen C, . Cyclic GMP-AMP is an endogenous second messenger in innate immune signaling by cytosolic DNA. Science 2013;339:826–30. 10.1126/science.1229963. PMC3855410 23258412

[21] Thrane S , Pedersen AM, Thomsen MBH, Kirkegaard T, Rasmussen BB, Duun-Henriksen AK, . A kinase inhibitor screen identifies Mcl-1 and Aurora kinase A as novel treatment targets in antiestrogen-resistant breast cancer cells. Oncogene 2015;34:4199–210. 10.1038/onc.2014.351. 25362855

[22] Krzyscik MA , Zakrzewska M, Sørensen V, Øy GF, Brunheim S, Haugsten EM, . Fibroblast growth factor 2 conjugated with monomethyl auristatin E inhibits tumor growth in a mouse model. Biomacromolecules 2021;22:4169–80. 10.1021/acs.biomac.1c00662. PMC8512659 34542998

[23] Schneider CA , Rasband WS, Eliceiri KW. NIH Image to ImageJ: 25 years of image analysis. Nat Methods 2012;9:671–5. 10.1038/nmeth.2089. PMC5554542 22930834

[24] Love MI , Huber W, Anders S. Moderated estimation of fold change and dispersion for RNA-seq data with DESeq2. Genome Biol 2014;15:550. 10.1186/s13059-014-0550-8. PMC4302049 25516281

[25] Bergamaschi A , Hjortland GO, Triulzi T, Sørlie T, Johnsen H, Ree AH, . Molecular profiling and characterization of luminal-like and basal-like in vivo breast cancer xenograft models. Mol Oncol 2009;3:469–82. 10.1016/j.molonc.2009.07.003. PMC5527532 19713161

[26] Cottu P , Marangoni E, Assayag F, de Cremoux P, Vincent-Salomon A, Guyader C, . Modeling of response to endocrine therapy in a panel of human luminal breast cancer xenografts. Breast Cancer Res Treat 2012;133:595–606. 10.1007/s10549-011-1815-5. 22002565

[27] Thennavan A , Beca F, Xia Y, Recio SG, Allison K, Collins LC, . Molecular analysis of TCGA breast cancer histologic types. Cell Genomics 2021;1:100067. 10.1016/j.xgen.2021.100067. PMC9028992 35465400

[28] Liu J , Lichtenberg T, Hoadley KA, Poisson LM, Lazar AJ, Cherniack AD, . An integrated TCGA pan-cancer clinical data resource to drive high-quality survival outcome analytics. Cell 2018;173:400–16.e11. 10.1016/j.cell.2018.02.052. PMC6066282 29625055

[29] Curtis C , Shah SP, Chin S-F, Turashvili G, Rueda OM, Dunning MJ, . The genomic and transcriptomic architecture of 2,000 breast tumours reveals novel subgroups. Nature 2012;486:346–52. 10.1038/nature10983. PMC3440846 22522925

[30] Rueda OM , Sammut SJ, Seoane JA, Chin SF, Caswell-Jin JL, Callari M, . Dynamics of breast-cancer relapse reveal late-recurring ER-positive genomic subgroups. Nature 2019;567:399–404. 10.1038/s41586-019-1007-8. PMC6647838 30867590

[31] Lee KW , Hong HR, Lim JS, Ko KP, Lee MG, Chi SG. XAF1 drives apoptotic switch of endoplasmic reticulum stress response through destabilization of GRP78 and CHIP. Cell Death Dis 2022;13:655. 10.1038/s41419-022-05112-0. PMC9334361 35902580

[32] Rosebeck S , Leaman DW. Mitochondrial localization and pro-apoptotic effects of the interferon-inducible protein ISG12a. Apoptosis 2008;13:562–72. 10.1007/s10495-008-0190-0. 18330707

[33] Sironi JJ , Ouchi T. STAT1-induced apoptosis is mediated by caspases 2, 3, and 7. J Biol Chem 2004;279:4066–74. 10.1074/jbc.M307774200. 14623896

[34] Sze A , Belgnaoui SM, Olagnier D, Lin R, Hiscott J, van Grevenynghe J. Host restriction factor SAMHD1 limits human T cell leukemia virus type 1 infection of monocytes via STING-mediated apoptosis. Cell Host Microbe 2013;14:422–34. 10.1016/j.chom.2013.09.009. 24139400

[35] Coombes RC , Badman PD, Lozano-Kuehne JP, Liu X, Macpherson IR, Zubairi I, . Results of the phase IIa RADICAL trial of the FGFR inhibitor AZD4547 in endocrine resistant breast cancer. Nat Commun 2022;13:3246. 10.1038/s41467-022-30666-0. PMC9187670 35688802

[36] Gong J , Mita AC, Wei Z, Cheng HH, Mitchell EP, Wright JJ, . Phase II study of erdafitinib in patients with tumors with FGFR amplifications: results from the NCI-MATCH ECOG-ACRIN trial (EAY131) subprotocol K1. JCO Precis Oncol 2024;8:e2300406. 10.1200/PO.23.00406. PMC11623914 38603651

[37] Sasaki S , Ishida T, Toyota M, Ota A, Suzuki H, Takaoka A, . Interferon-α/β and anti-fibroblast growth factor receptor 1 monoclonal antibody suppress hepatic cancer cells in vitro and in vivo. PLoS One 2011;6:e19618. 10.1371/journal.pone.0019618. PMC3090414 21573021

[38] Ma F , Li B, Yu Y, Iyer SS, Sun M, Cheng G. Positive feedback regulation of type I interferon by the interferon-stimulated gene STING. EMBO Rep 2015;16:202–12. 10.15252/embr.201439366. PMC4328747 25572843

[39] Lv QM , Lei HM, Wang SY, Zhang KR, Tang YB, Shen Y, . Cancer cell-autonomous cGAS-STING response confers drug resistance. Cell Chem Biol 2023;30:591–605.e4. 10.1016/j.chembiol.2023.05.005. 37263275

[40] Li Q , Song Q, Ma L, Kang K, Zhu X, Li Y, . The cGAS-STING pathway in cancer: friend or foe. Cell Death Dis 2026;17:374. 10.1038/s41419-026-08607-2. PMC13040067 41881950

