# Supplementary material for: FGFR1 Suppresses STING-Mediated Interferon Response in Endocrine Therapy–Resistant Breast Cancer
Source: Cancer Res Commun. 2026 Sep 21;6(9):2220–31. doi: 10.1158/2767-9764.CRC-25-0821 (PMC13591376; doi:10.1158/2767-9764.CRC-25-0821)
Supplement: Supplementary file 1 [file crc-25-0821_supplementary_figure_1_suppsf1.pdf]

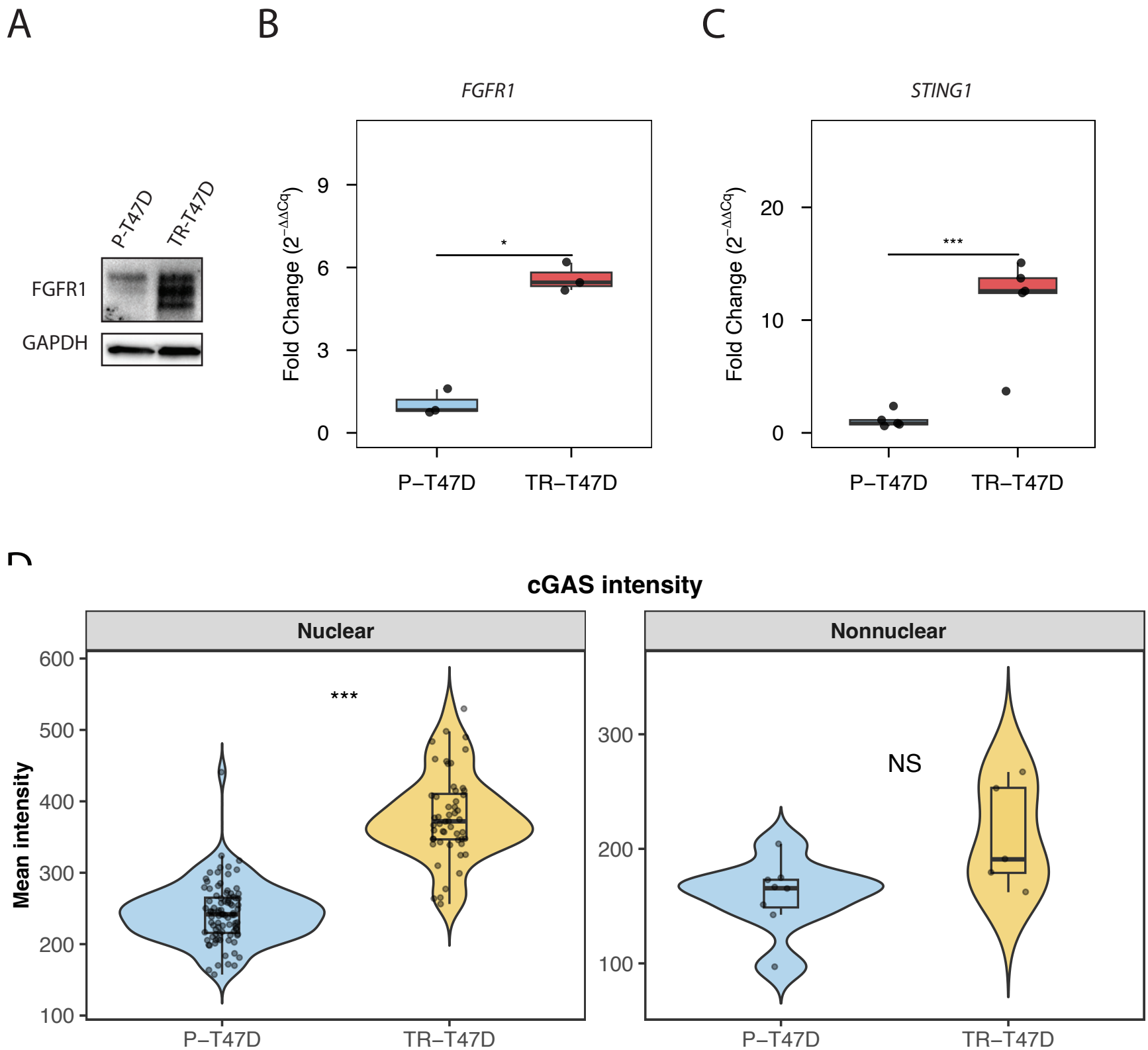

Supplementary Figure S1

(A) Western blot shows FGFR1 protein levels in P-T47D and TR-T47D cells. GAPDH as loading control. **(B–C)** Relative expression of *FGFR1* and *STING1* respectively, in P-T47D and TR-T47D cells, measured by RT-qPCR and normalized to *ACTB* and *RPLP1*. Fold change was calculated relative to the geometric mean of ΔCq of the P-T47D condition. Boxplots show the median and IQR with whiskers extending to the most extreme data point within 1.5 × IQR of the box edges; individual points represent biological replicates. **(D)** Mean cGAS immunofluorescence intensity in P-T47D and TR-T47D cells in nuclear (left; each point = one nucleus) and non-nuclear (right; each point = one image) compartments. Violin plots show data distribution with overlaid boxplots displaying the median and IQR; whiskers extend to the most extreme data point within 1.5 × IQR of the box edges. P-values are indicated by *asterisks* (NS, non-significant, * $P < 0.05$, *** $P < 0.001$).
