# Supplementary material for: FGFR1 Suppresses STING-Mediated Interferon Response in Endocrine Therapy–Resistant Breast Cancer
Source: Cancer Res Commun. 2026 Sep 21;6(9):2220–31. doi: 10.1158/2767-9764.CRC-25-0821 (PMC13591376; doi:10.1158/2767-9764.CRC-25-0821)
Supplement: Supplementary file 2 [file crc-25-0821_supplementary_figure_2_suppsf2.pdf]

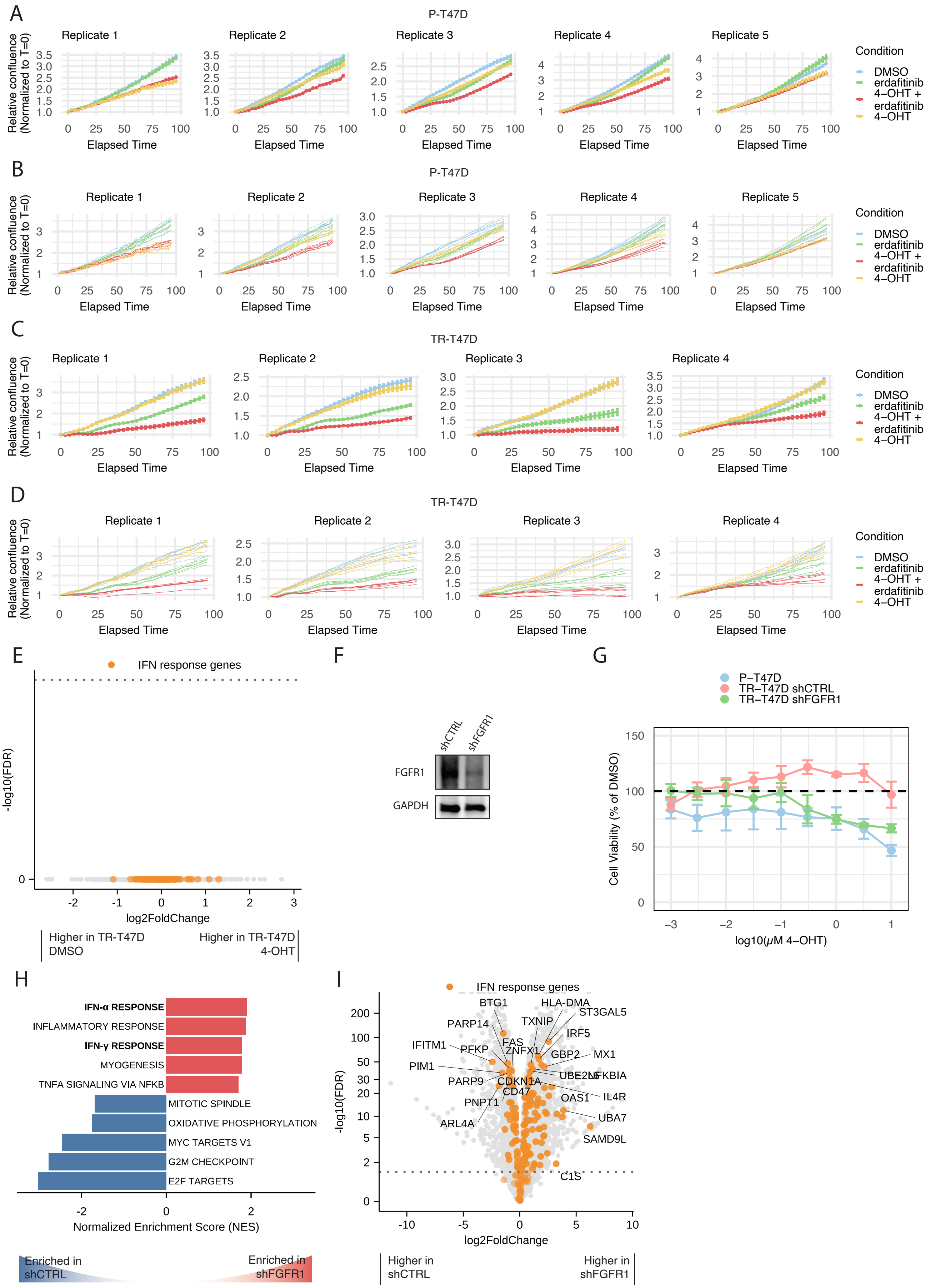

Supplementary Figure S2

(A–B) Growth curves of P-T47D cells treated with DMSO, 4-OHT, erdafitinib, or the combination of 4-OHT and erdafitinib. **(A)** Mean relative confluence ± standard error (SE), normalized to the starting timepoint (T = 0), shown separately for five biological replicates. **(B)** Individual growth curves for all technical replicates within each biological replicate shown in panel A, illustrating intra-replicate variability. **(C–D)** Growth curves of TR-T47D cells treated DMSO, 4-OHT, erdafitinib, or the combination of 4-OHT and erdafitinib. **(C)** Mean relative confluence ± SE, normalized to the starting timepoint (T = 0), shown separately for four biological replicates. **(D)** Individual growth curves for all technical replicates within each biological replicate shown in panel C, illustrating intra-replicate variability. **(E)** Differentially expressed genes in TR-T47D cells treated with 4-OHT versus DMSO. The dashed horizontal line indicates the threshold for statistical significance (FDR < 0.05). **(F)** Western blot shows FGFR1 protein levels in TR-T47D^{shCTRL} and TR-T47D^{shFGFR1} cells. GAPDH as loading control. **(G)** Cell viability was measured using the MTS assay across a range of 4-OHT concentrations in P-T47D cells, TR-T47D^{shFGFR1} and TR-T47D^{shCTRL} cells. Data are shown as mean viability ± standard error (SE) relative to DMSO-treated controls. The x-axis represents the log10-transformed 4-OHT concentration (µM); the dashed line indicates 100% viability. **(H)** GSEA of Hallmark pathways in TR-T47D^{shFGFR1} versus TR-T47D^{shCTRL} cells. Bars represent normalized enrichment scores for the five most significantly enriched gene sets in each direction (FDR < 0.05). **(I)** Differentially expressed genes in TR-T47D^{shFGFR1} cells versus TR-T47D^{shCTRL}. The dashed horizontal line indicates the threshold for statistical significance (FDR < 0.05).
