# Supplementary material for: FGFR1 Suppresses STING-Mediated Interferon Response in Endocrine Therapy–Resistant Breast Cancer
Source: Cancer Res Commun. 2026 Sep 21;6(9):2220–31. doi: 10.1158/2767-9764.CRC-25-0821 (PMC13591376; doi:10.1158/2767-9764.CRC-25-0821)
Supplement: Supplementary file 3 [file crc-25-0821_supplementary_figure_3_suppsf3.pdf]

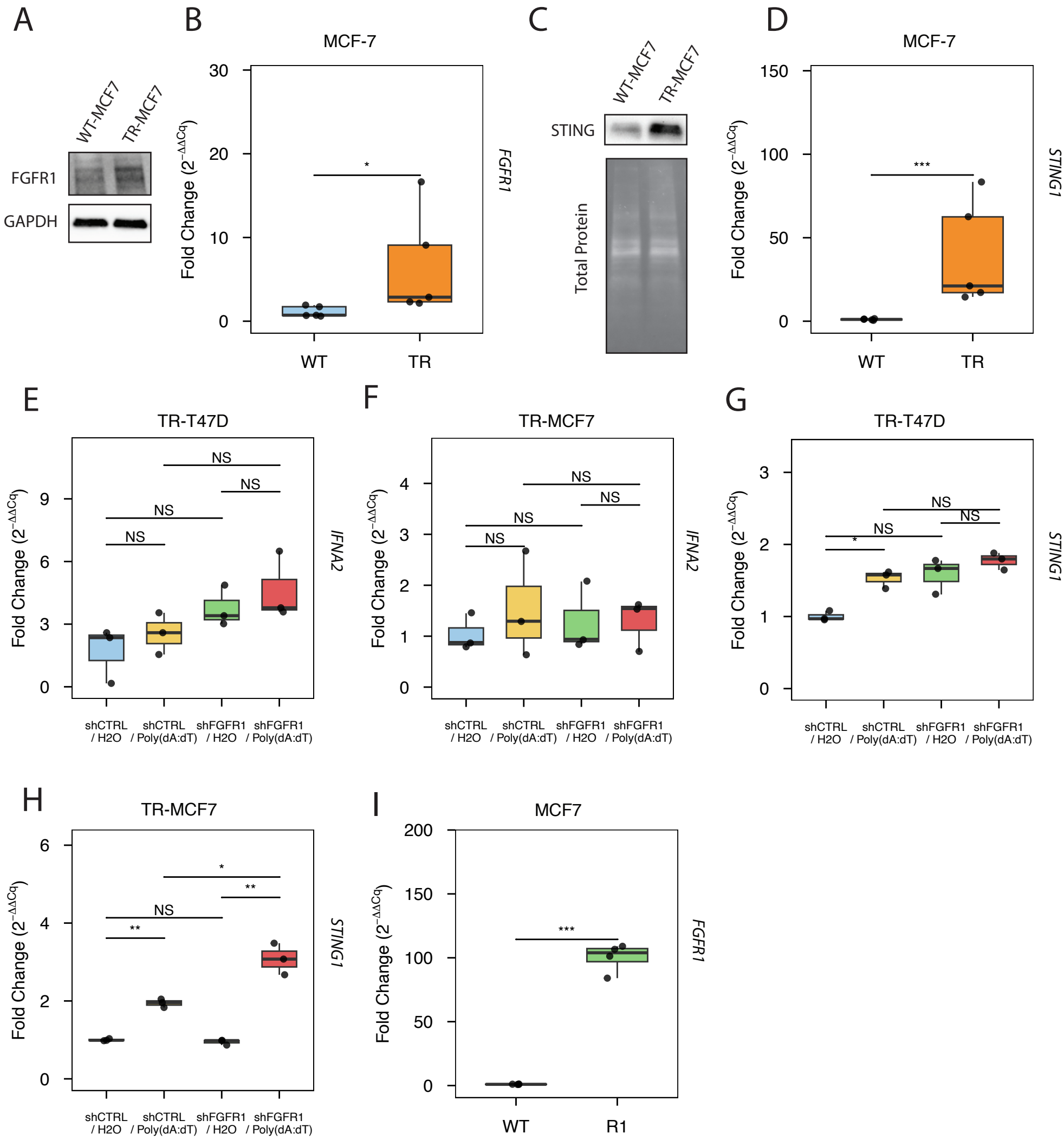

Supplementary Figure S3

(A) Western blot shows FGFR1 protein levels in WT-MCF7 and TR-MCF7 cells. GAPDH was as loading control. **(B)** Relative expression of *FGFR1* in WT-MCF7 and TR-MCF7 cells, measured by RT–qPCR and normalized to *ACTB* and *RPLP1*. Fold change was calculated relative to the geometric mean of ΔCq of the MCF7-WT condition. **(C)** Western blot shows STING protein levels in WT-MCF7 and TR-MCF7. Total protein as loading control. **(D)** Relative expression of *STING1* in WT-MCF7 and TR-MCF7 cells measured by RT–qPCR and normalized to *ACTB* and *RPLP1*. Fold change was calculated relative to the geometric mean of ΔCq of the MCF7-WT condition. **(E–H)** Relative expression of *IFNA2* **(E-F)** and *STING1* **(G–H)** in TR-T47D^{shCTRL}/TR-T47D^{shFGFR1} cells and TR-MCF7^{shCTRL}/TR-MCF7^{shFGFR1} cells respectively, treated with either H_2O or poly(dA:dT), measured by RT–qPCR and normalized to *GAPDH* and *RPLP1*. Fold change was calculated relative to the geometric mean of ΔCq of the shCTRL/H_2O condition. **(I)** Relative expression of *FGFR1* in WT-MCF7 and R1-MCF7 cells, measured by RT-qPCR and normalized to *HPRT1*. Fold change was calculated relative to the geometric mean of ΔCq of the WT-MCF7 condition. Boxplots show the median and IQR with whiskers extending to the most extreme data point within 1.5 × IQR of the box edges; individual points represent biological replicates. *P*-values are indicated by *asterisks* (NS, non-significant, * $P < 0.05$, ** $P < 0.01$), *** $P < 0.001$).
