# Supplementary material for: FGFR1 Suppresses STING-Mediated Interferon Response in Endocrine Therapy–Resistant Breast Cancer
Source: Cancer Res Commun. 2026 Sep 21;6(9):2220–31. doi: 10.1158/2767-9764.CRC-25-0821 (PMC13591376; doi:10.1158/2767-9764.CRC-25-0821)
Supplement: Supplementary file 4 [file crc-25-0821_supplementary_figure_4_suppsf4.pdf]

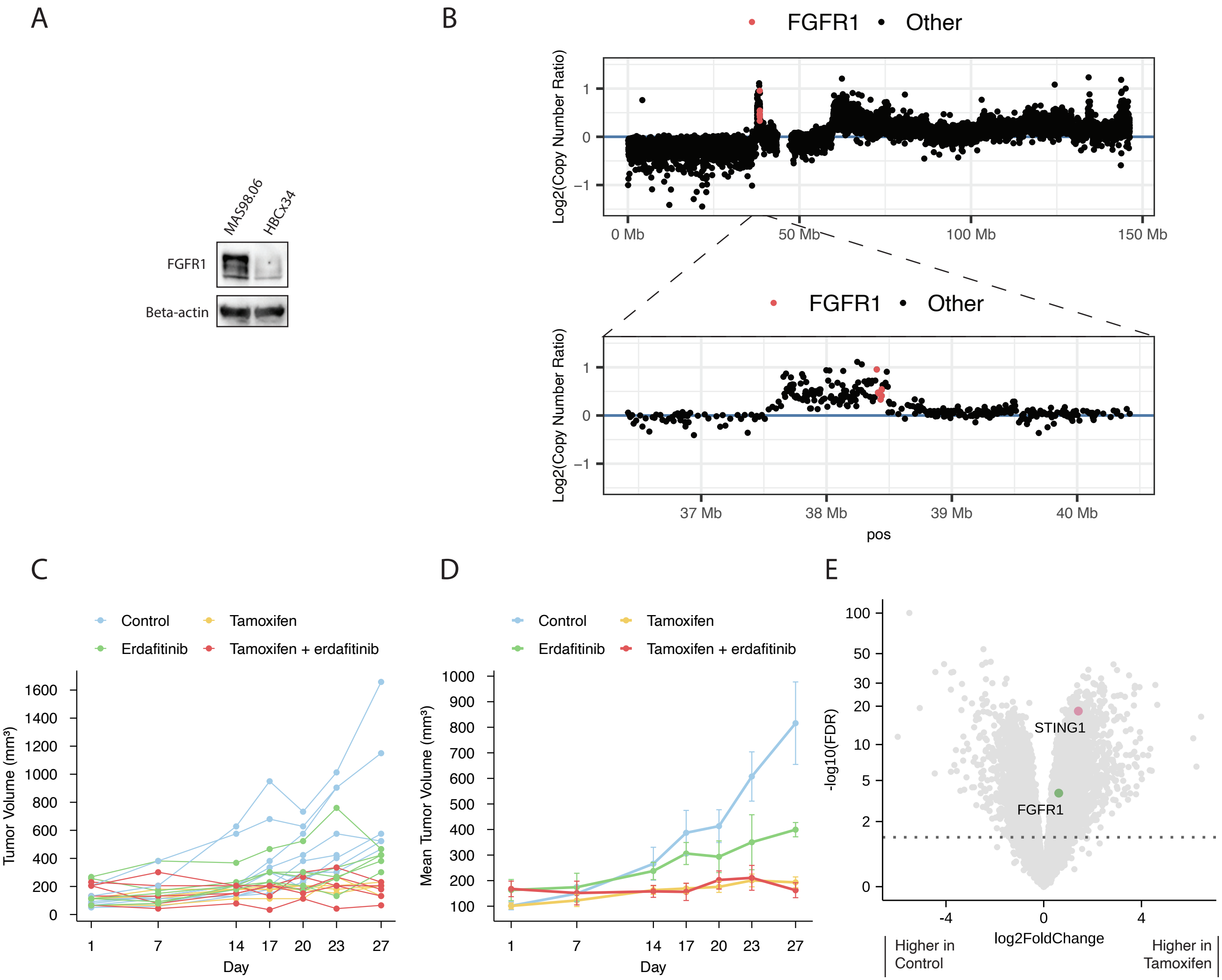

Supplementary Figure S4

(A) Western blot shows FGFR1 protein levels in two luminal breast cancer PDX models MAS98.06 (FGFR1-amplified) and HBCx34 (FGFR1 non-amplified). β-actin as loading control. **(B)** Array-based comparative genomic hybridization (aCGH) $\log_2$-ratio profile of chromosome 8 in MAS98.06. Each point represents an individual probe on a CGH microarray. Red points indicate probes mapping to the *FGFR1* locus (chr8: 38.17–38.58 Mb), highlighting a focal amplification. The top panel displays the full chromosome 8 profile, while the lower panel shows a zoomed-in view of the genomic region surrounding the *FGFR1* gene. The y-axis represents $\log_2$(test/reference) signal intensity, where values above zero indicate copy number gain and values below zero indicate loss. The x-axis indicates relative position along chromosome 8. **(C)** Individual tumor growth of MAS98.06 PDX (control, tamoxifen, erdafitinib, or the combination tamoxifen + erdafitinib treated). Tumor volumes were measured at multiple time points, and each line represents a single tumor. Only tumors with initial volume between 50 and 300 mm^3 were included in the analysis. **(D)** Mean tumor volume for the MAS98.06 tumors shown in panel C. Lines represent the average tumor volume for each treatment group, and error bars indicate the standard error of the mean (SEM) at each time point. **(E)** Differentially expressed genes in tamoxifen versus control treated MAS98.06 tumors. *FGFR1* and *STING1* are indicated. The dashed horizontal line indicates the threshold for statistical significance ($FDR < 0.05$).
