# Supplementary material for: FGFR1 Suppresses STING-Mediated Interferon Response in Endocrine Therapy–Resistant Breast Cancer
Source: Cancer Res Commun. 2026 Sep 21;6(9):2220–31. doi: 10.1158/2767-9764.CRC-25-0821 (PMC13591376; doi:10.1158/2767-9764.CRC-25-0821)
Supplement: Supplementary file 5 [file crc-25-0821_supplementary_figure_5_suppsf5.pdf]

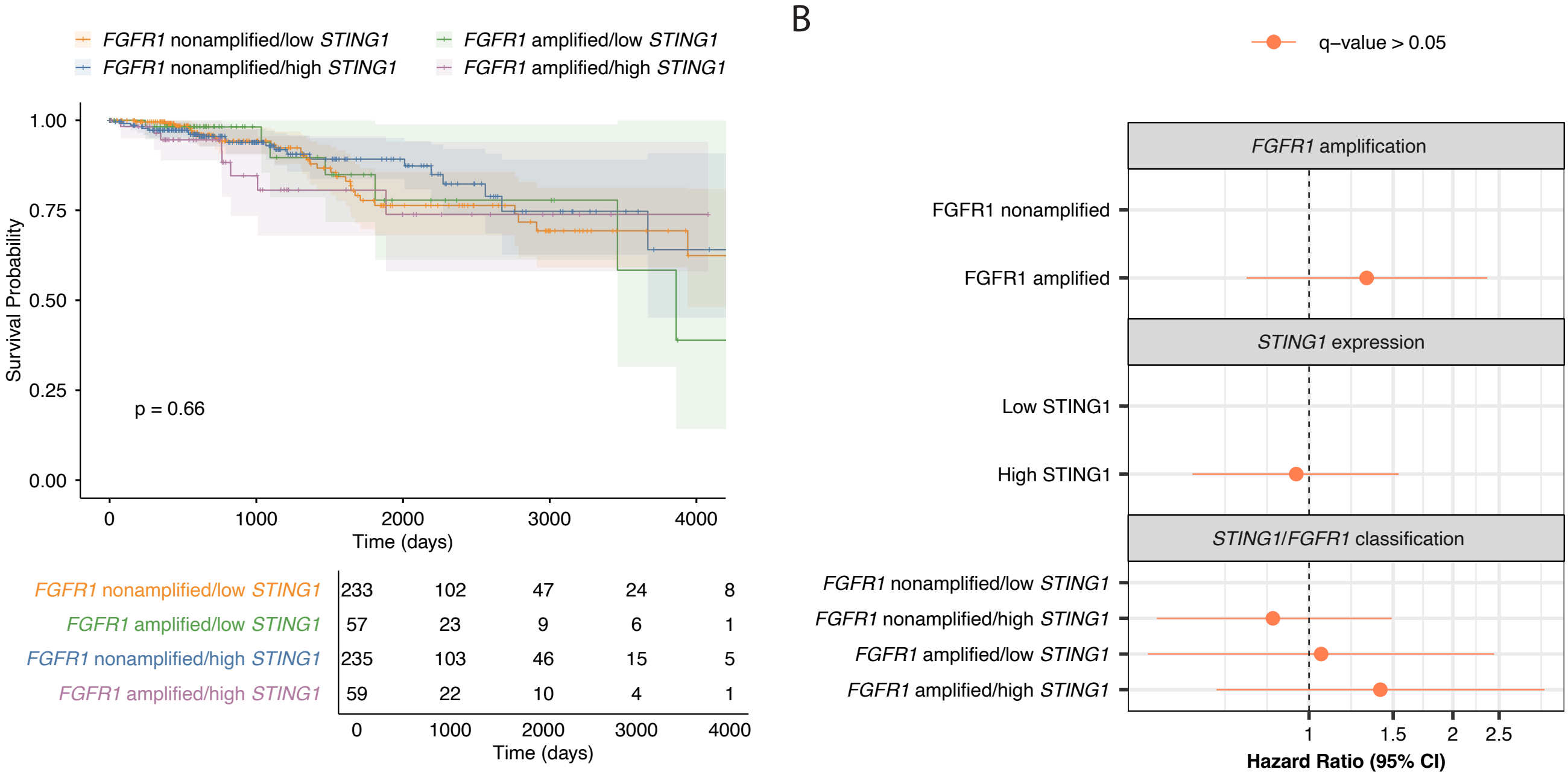

Supplementary Figure S5

(A) Disease-free survival (DFS) stratified by combined FGFR1 amplification and *STING1* expression status in ER+/HER2– breast tumors from the TCGA-BRCA cohort. FGFR1 amplification was defined as copy number ≥ 6. *STING1* expression was dichotomized at the median into "High *STING1*" and "Low *STING1*". Curves are truncated at 4,000 days due to the low number of patients at risk beyond this point. Log-rank *P*-values and 95% confidence intervals are shown. **(B)** Univariate Cox regression analysis of DFS in ER+/HER2– breast tumors from the TCGA-BRCA cohort, stratified by FGFR1 amplification and *STING1* expression. Hazard ratios (HRs) and 95% confidence intervals (CIs) are shown. FGFR1 amplification was defined as copy number ≥ 6, and *STING1* expression was dichotomized at the median into "High *STING1*" and "Low *STING1*" The four resulting subgroups are the same as those shown in panel A. Hazard ratios are displayed on a log10 scale, with the reference group set as "FGFR1 Non-amplified / Low *STING1*". Point shapes indicate statistical significance following FDR correction (q-value < 0.05).
